# Transcriptomics analysis of *Toxoplasma gondii*-infected mouse macrophages reveals coding and noncoding signatures in the presence and absence of MyD88

**DOI:** 10.1186/s12864-021-07437-0

**Published:** 2021-02-23

**Authors:** Kayla L. Menard, Lijing Bu, Eric Y. Denkers

**Affiliations:** Center for Evolutionary and Theoretical Immunology and Department of Biology, University of New Mexico, Albuquerque, NM USA

**Keywords:** *Toxoplasma gondii*, Parasite, Macrophages, Noncoding RNA, lncRNA, MyD88

## Abstract

**Background:**

*Toxoplasma gondii* is a globally distributed protozoan parasite that establishes life-long asymptomatic infection in humans, often emerging as a life-threatening opportunistic pathogen during immunodeficiency. As an intracellular microbe, *Toxoplasma* establishes an intimate relationship with its host cell from the outset of infection. Macrophages are targets of infection and they are important in early innate immunity and possibly parasite dissemination throughout the host. Here, we employ an RNA-sequencing approach to identify host and parasite transcriptional responses during infection of mouse bone marrow-derived macrophages (BMDM). We incorporated into our analysis infection with the high virulence Type I RH strain and the low virulence Type II strain PTG. Because the well-known TLR-MyD88 signaling axis is likely of less importance in humans, we examined transcriptional responses in both MyD88^+/+^ and MyD88^−/−^ BMDM. Long noncoding (lnc) RNA molecules are emerging as key regulators in infection and immunity, and were, therefore, included in our analysis.

**Results:**

We found significantly more host genes were differentially expressed in response to the highly virulent RH strain rather than with the less virulent PTG strain (335 versus 74 protein coding genes for RH and PTG, respectively). Enriched in these protein coding genes were subsets associated with the immune response as well as cell adhesion and migration. We identified 249 and 83 non-coding RNAs as differentially expressed during infection with RH and PTG strains, respectively. Although the majority of these are of unknown function, one conserved lncRNA termed mir17hg encodes the mir17 microRNA gene cluster that has been implicated in down-regulating host cell apoptosis during *T. gondii* infection. Only a minimal number of transcripts were differentially expressed between MyD88 knockout and wild type cells. However, several immune genes were among the differences. While transcripts for parasite secretory proteins were amongst the most highly expressed *T. gondii* genes during infection, no differentially expressed parasite genes were identified when comparing infection in MyD88 knockout and wild type host BMDM.

**Conclusions:**

The large dataset presented here lays the groundwork for continued studies on both the MyD88-independent immune response and the function of lncRNAs during *Toxoplasma gondii* infection.

**Supplementary Information:**

The online version contains supplementary material available at 10.1186/s12864-021-07437-0.

## Background

The intracellular apicomplexan *Toxoplasma gondii* is a globally distributed parasitic microorganism infecting both humans and animals. In humans alone, *Toxoplasma* is conservatively estimated to be present in over a billion individuals [1]. After ingestion of tissue cysts or oocysts, an acute phase commences characterized by parasite dissemination throughout the host as rapidly dividing tachyzoites. This is followed by establishment of latent infection, in which tachyzoites differentiate into slowly replicating bradyzoites that form cysts in tissues of the central nervous system and skeletal muscle [2, 3]. Latent, or chronic, infection is asymptomatic in most cases, but the parasite may reactivate in immunocompromised populations leading to life-threatening disease [4]. Primary infection during pregnancy can lead to major birth defects and sequelae of infection later in life [5].

*Toxoplasma* is well known for its ability to stimulate strong Th1 immunity that has as its origin early production of IL-12 by dendritic cells [6, 7]. The IFN-γ produced during infection confers resistance to the parasite, and indeed this cytokine is central in the ability to survive acute *Toxoplasma* infection [8]. While protective, IFN-γ production can result in host pathology if not appropriately regulated by counter-inflammatory cytokines such as IL-10 [9, 10]. A major function of IFN-γ is to elicit inflammatory macrophages that are major anti-microbial effectors during in vivo infection [11–13]. Paradoxically, macrophages along with dendritic cells also serve as early cells targeted for infection, and it has been suggested that they act as Trojan horses to enable establishment of *T. gondii* in the host [14–18]. For these reasons, macrophages are an especially important cell type to study both the host immune response and *T. gondii* behavior during intracellular infection.

Substantial work in mouse models has revealed an important role for Toll-like receptor (TLR) and the adaptor molecule MyD88 in innate immune recognition of *T. gondii* [19, 20]*.* The invasion-associated parasite protein profilin functions as a ligand for TLR11 and TLR12, initiating MyD88-dependent immunity [21–24]. Given the central role of the MyD88 protein in the early innate immune response in mice to *T. gondii* infection, it is important to understand how deletion of MyD88 impacts transcription of downstream immune genes in infected cells. In humans, the basis of immune recognition is less clear because TLR11 is present as a pseudogene and TLR12 is absent from the genome [25]. Furthermore, a study of a pediatric population with an autosomal recessive MyD88 deficiency revealed that these individuals retain resistance to all but a minimal number of pyogenic bacterial infections [26, 27]. Thus, determining MyD88-independent responses to infection with *Toxoplasma* and other microbial pathogens is an important avenue of investigation in both humans and mice.

We therefore employed RNA sequencing (RNA-seq) to determine the transcriptome of MyD88^+/+^ and MyD88^−/−^ bone marrow-derived macrophages (BMDM) following infection with *T. gondii*. In addition to yielding information on protein coding responses, RNA-seq provides insight into responses of long noncoding RNA (lncRNA), defined as transcripts greater than 200 nucleotides with no protein coding potential. lncRNAs are widely involved in gene regulation, and their study is an emerging area of interest in infection and immunology [28–31].

Our approach involved infection with high virulence (Type I strain RH) and low virulence (Type II strain PTG) isolates of *Toxoplasma*. Amongst Type II strains, some differences in the intensity of cytokine responses have been noted with different isolates but we employed a strain that has been extensively used in previous studies [32]. In mice, Type I strains induce a hyperinflammatory cytokine response rapidly culminating in host death. The immune response is more restrained during Type II infection, enabling host survival and parasite establishment of latent infection [33, 34]. In vitro studies have revealed that infection with these strains activates partially nonoverlapping host signaling pathways leading to distinct responses. For example, infection with Type I parasites triggers strong and sustained activation of STAT3 and STAT6 resulting in the generation of macrophages with an M2 phenotype [35, 36]. Type II infection triggers NFκB activation and robust IL-12 production [37]. The present study provides important information on global transcriptional changes in macrophages infected with these two *Toxoplasma* strains in the presence and absence of MyD88.

In addition to examining the transcriptional changes in macrophages, use of RNA-seq technology enabled us to simultaneously harvest data on the transcriptomes of high and low virulence *Toxoplasma* during initial stages of intracellular infection. This allowed us to compare gene expression differences between *T. gondii* strains, as well as examine differences in parasite gene expression when infecting MyD88^+/+^ versus MyD88^−/−^ macrophages. Together, this dataset provides a host and parasite genomic framework for understanding the interactome that emerges during intracellular infection with *Toxoplasma.*

## Results

### Dual RNA sequencing of *Toxoplasma*-infected macrophages identifies host and parasite transcripts

**Fig. 1:**
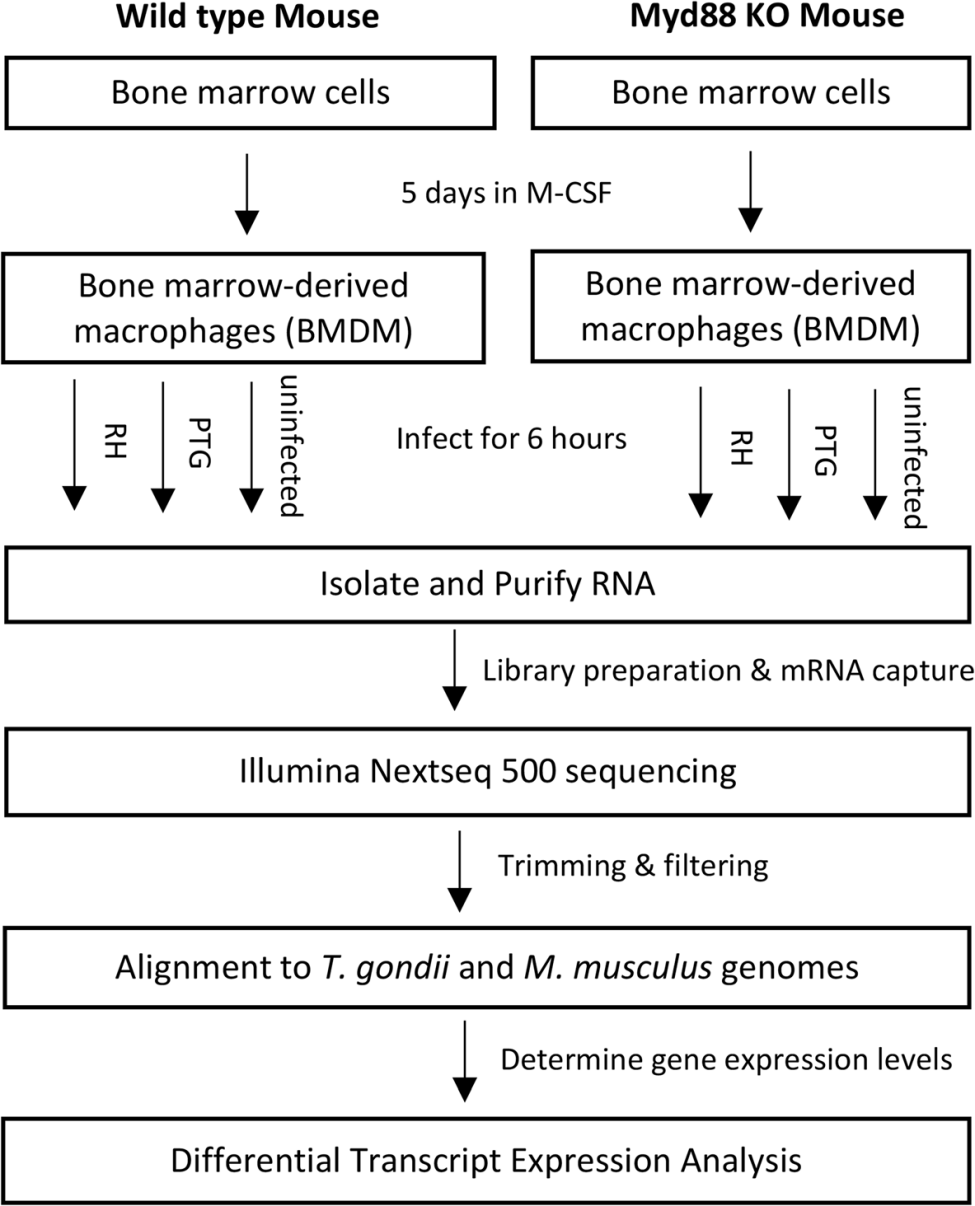
Flow chart demonstrating the steps taken to identify differentially expressed transcripts during *T. gondii* infection of wild type and MyD88 KO mouse macrophages

**Fig. 2:**
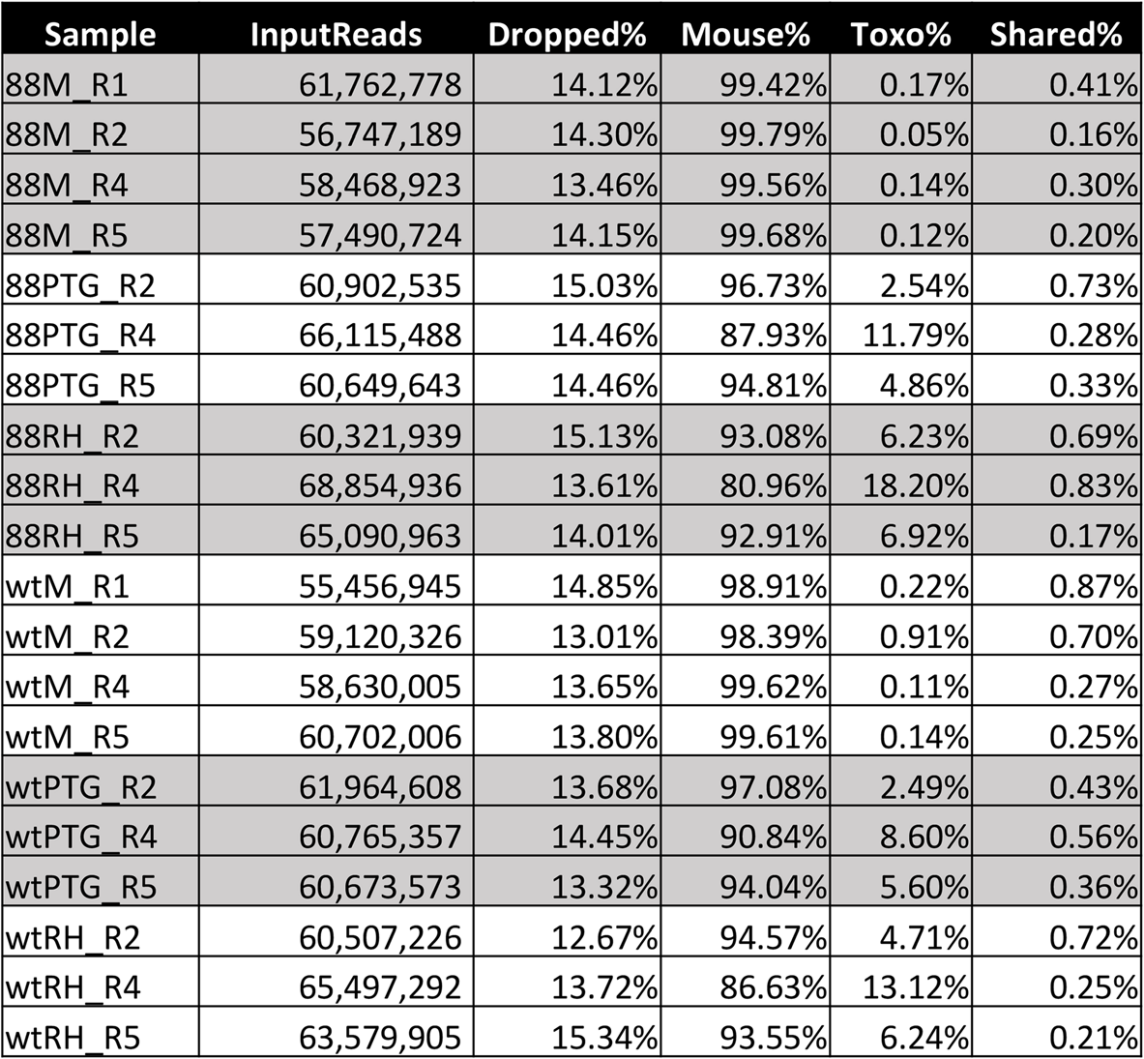
Overview of RNA-sequencing reads mapping to both mouse and *Toxoplasma* genomes. A total of 20 RNA samples were submitted for sequencing, and characteristics of each sample are provided here. Column 1 denotes the sample name. 88, MyD88 KO BMDM; wt, wild type BMDM; M, noninfected macrophages; RH, macrophages infected with Type I RH strain *Toxoplasma*; PTG, samples infected with *T. gondii* Type II PTG strain. The numbers indicate independent biological replicates. InputReads (Column 2) denotes the number of reads obtained for each sample. Dropped % (Column 3) indicates the percent of input reads deemed low-quality and dropped. Mouse % (Column 4) is the percent of high-quality reads that mapped to the mouse genome. Toxo % (Column 5) denotes the percent of high-quality reads that mapped to the *T. gondii* genome. Shared % (Column 6) indicates the percent of high-quality reads mapping to both mouse and *T. gondii* genomes

We infected wild type and MyD88 knockout (KO) bone marrow-derived macrophages with both Type I (RH) and Type II (PTG) *Toxoplasma* tachyzoites, then collected samples 6 h later for high throughput RNA-seq (Fig. 1). We selected 6 h for our analysis because this time point occurs prior to the first parasite mitotic division, controlling for differences in replication rate between Type I and Type II strains. This time also enabled us to determine the earliest macrophage responses to infection. We used a multiplicity of infection (MOI) of 4:1 or 5:1. The percent infection, measured via fluorescence microscopy at 6 h over multiple biological replicates, ranged from 70.4–95.7%. Sequencing was performed on 4 biological replicates for uninfected samples and 3 biological replicates for infected samples. We mapped mouse reads to GENCODE version M21, a database containing sequences for 58,899 protein-coding transcripts and approximately 30,462 lncRNA transcripts. We mapped *Toxoplasma* reads to ME49 strain sequences in the ToxoDB database. As expected, mouse sequences comprised the vast majority of reads in infected samples (Fig. 2). The percentage of reads mapping to the *T. gondii* genome ranged from 2.49 to 18.20% between samples. The variability in percentage of mapped reads between replicates correlated only weakly, at best, with percent infection measured via fluorescence microscopy (RH R^2^ = 0.18; PTG R^2^ = 0.01) but did correlate more strongly with the number of parasites per infected cell (RH R^2^ = 0.15; PTG R^2^ = 0.68). Factors in addition to the number of parasites present likely contribute to the variability in the number of parasite transcripts produced. The background level of *T. gondii* reads in uninfected samples was determined to not affect the parasite gene expression results presented herein and likely represent mis-mapping of mouse genes, since housekeeping genes were among the top *Toxoplasma* genes in uninfected samples. Principal component analysis (PCA) plots of mapped mouse reads demonstrate that the treatment (in this case parasite strain) accounted for data variability, but also that the biological replicate contributed substantially to the variability observed (Additional files 1, 2 and 3). PCA plots of mapped *Toxoplasma* reads demonstrate that the parasite strain largely accounted for data variability (Additional files 4, 5).

### Type I strain parasites trigger stronger protein-coding gene expression effects compared to type II parasites

**Fig. 3:**
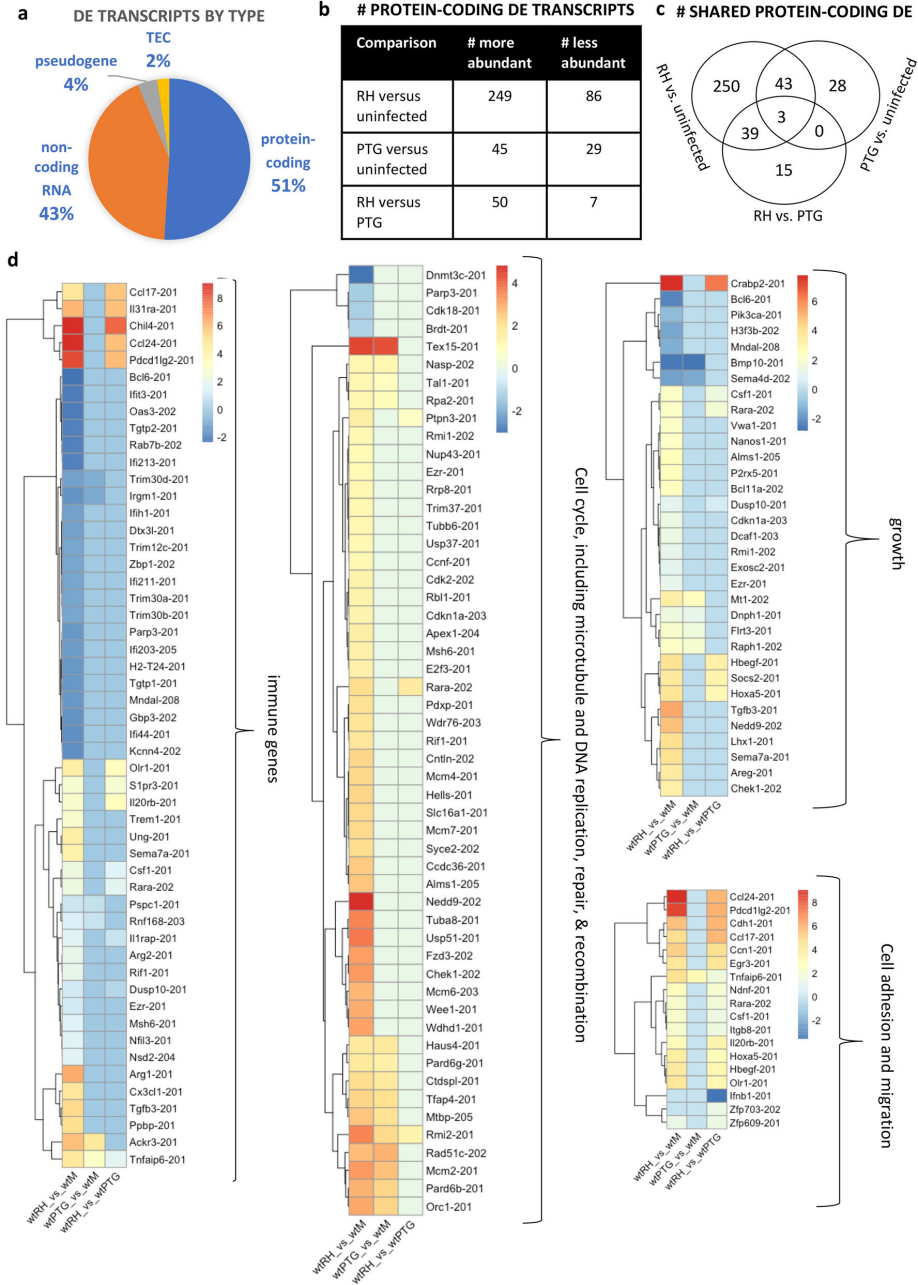
A much greater number of protein-coding genes are differentially expressed during infection with the highly virulent *Toxoplasma* RH strain than with the less virulent PTG strain. Wild type BMDM were infected with either the highly virulent RH strain or the less-virulent PTG strain, and 6 h later RNA was isolated for sequencing. Differentially expressed mouse transcripts were identified based on statistical significance (PPDE greater than 0.95) and a fold change of greater or less than 2. **a** Classification of differentially expressed mouse transcripts as either protein-coding, non-coding, pseudogene, or TEC (To be Experimentally Confirmed). **b** Total number of protein-coding transcripts of higher or lower abundance during infection. **c** Venn diagrams of differentially expressed protein-coding transcripts showing shared and unique expression patterns between infection strains. **d** Heat maps displaying trends among functionally related genes. Experiments were performed in at least triplicate with BMDM preparations from separate mice

We defined differentially expressed (DE) transcripts using a *p*-value of equal or less than 0.05 and a fold change of 2 or greater. Among the wild type mouse samples, DE transcripts were primarily protein-coding (51%) and non-coding (43%), with pseudogenes and TEC (To be Experimentally Confirmed) comprising 4 and 2% of the hits respectively (Fig. 3a). A complete listing of DE transcripts for the three wild type comparisons (RH vs uninfected, PTG vs uninfected, and RH vs PTG) is shown in Additional file 6.

Among the protein-coding sequences, substantially more DE transcripts were differentially expressed with the Type I RH infection versus with the less-virulent Type II PTG infection (335 and 74, respectively). This indicates that RH has a stronger impact on the host macrophages relative to PTG. 57 transcripts were differentially expressed between RH and PTG, including previously known immune genes ccl24, csf1, socs2 and ccl17 (Fig. 3b and Additional file 6). Venn diagrams reveal that 46 DE transcripts (62%) are shared between RH and PTG infection (Fig. 3c).

Heat maps demonstrate that many genes related to the immune response, cell cycle, DNA replication, DNA recombination, DNA repair, growth, cell adhesion, and cell migration were differentially expressed during *T. gondii* infection (Fig. 3d). Numerous immune genes were of higher or lower abundance during RH infection, confirming that activation and suppression of immunity during *Toxoplasma* infection extends to the cellular level (Fig. 3d). In confirmation of previous studies [35, 36, 38], immune-related genes Arg1 and ccl17 were more abundant in RH versus uninfected cells. Many cell adhesion and migration genes were more abundant in both the RH versus uninfected and RH versus PTG comparisons. Many genes related to the cell cycle were differentially expressed in both RH versus uninfected and PTG versus uninfected. The DE genes for cell cycle include several genes relating to microtubule organizing center and DNA replication, recombination, and repair. This is of interest because *Toxoplasma* is thought to co-opt microtubules for its own survival [39, 40]. Many cell growth genes were of higher abundance, particularly for RH versus uninfected. Gene ontology analysis and KEGG pathway analysis results support the data shown in the heat maps (Additional file 7). In addition to the functional categories displayed in the heat maps, gene ontology analysis indicates that metabolic processes are also strongly differentially expressed in the RH strain (Additional file 7). Additional heat maps with each replicate displayed individually reveal that sample wtRH_4 had stronger effects than other replicates but the same overall trends (Additional Files 8 and 9).

### Many noncoding transcripts are differentially expressed during infection of wild type BMDM

**Fig. 4:**
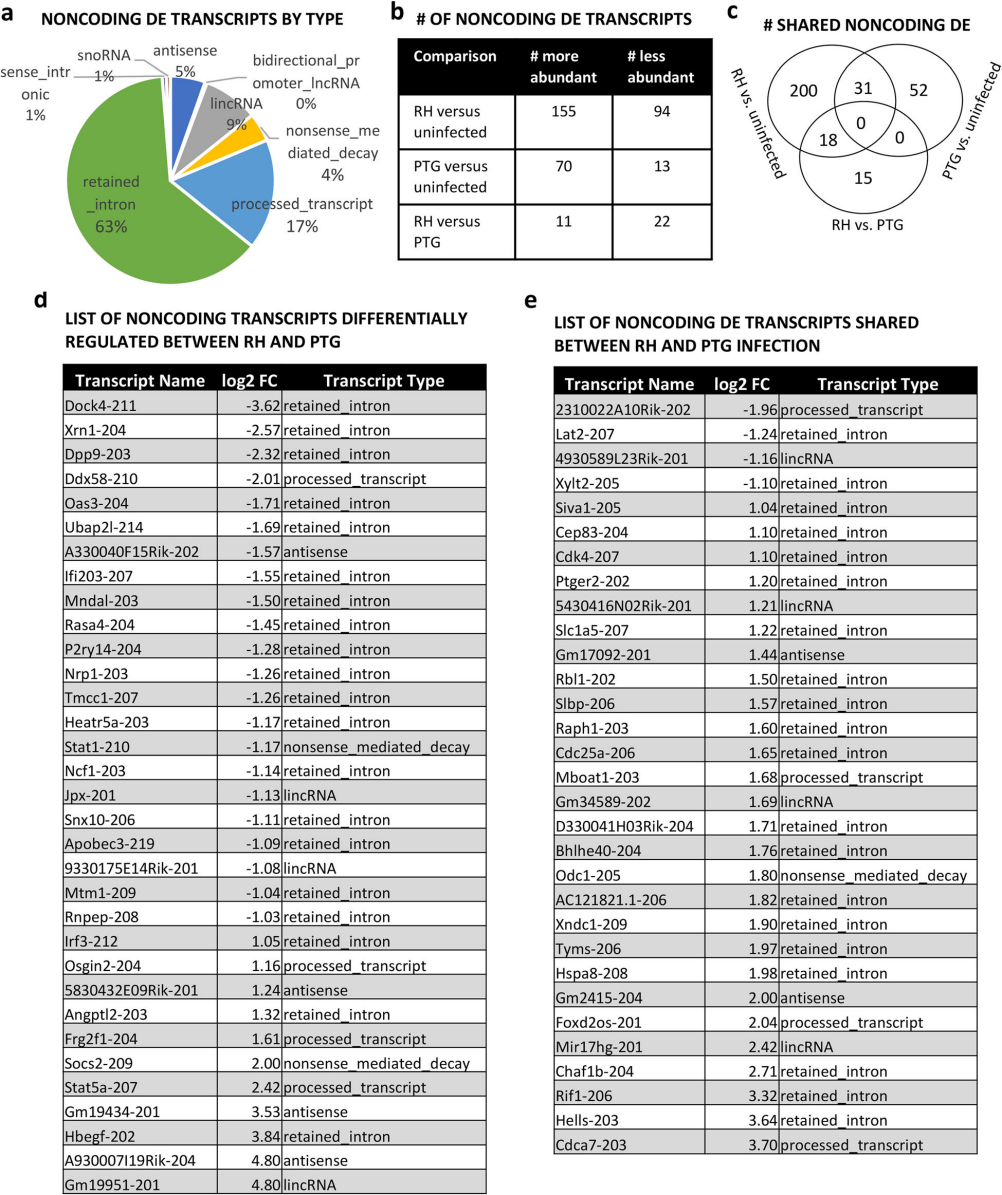
Many non-coding transcripts are differentially expressed during infection with *Toxoplasma*. Wild type BMDM were infected with either the highly virulent RH strain or the less-virulent PTG strain, and 6 h later RNA was isolated for sequencing. Differentially expressed mouse non-coding transcripts were identified based on statistical significance (PPDE greater than 0.95) and a fold change of greater or less than 2. **a** Classification of differentially expressed mouse non-coding transcripts by type. **b** Total number of non-coding transcripts of higher or lower abundance during infection. **c** Venn diagrams of differentially expressed non-coding transcripts revealing shared and unique expression patterns between infection strains. **d** List of all noncoding transcripts differentially expressed between RH and PTG. **e** List of all noncoding differentially expressed transcripts shared between RH and PTG infection. Experiments were performed in at least triplicate with BMDM from separate mice

Among the mouse reads mapped to GENCODE/Ensembl, we analyzed the noncoding transcripts separately from the protein-coding transcripts. The majority (63%) of the differentially expressed noncoding transcripts identified in wild type BMDM are classified in GENCODE/Ensembl as *retained_intron* noncoding RNAs, defined as alternatively spliced transcripts believed to contain intronic sequences relative to other coding transcripts of the same gene (Fig. 4a). Many of these intronic transcripts may be pieces of pre-mRNAs or excised introns that are targeted for degradation. Since we cannot rule out a function for them as regulatory RNAs, they were included in this analysis. 17% of the noncoding RNAs are classified as *processed_transcript*, a general term for a gene/transcript that lacks an open reading frame. Nine percent of the noncoding RNAs fell into the category of *lincRNA*, defined as long intergenic noncoding RNA. Four percent are classified as *nonsense_mediated_decay*, transcripts that contain sequences tagging them for destruction. While not specifically defined as noncoding in Ensembl, *nonsense_mediated_decay* transcripts could possibly have functions as noncoding RNAs, so were included in the analysis. Five percent are classified as *antisense* or “transcripts that overlap the genomic span of a protein-coding locus on the opposite strand”. *Bidirectional_promoter lncRNAs*, *sense_intronic*, and *snoRNA* comprised 1% or less of the noncoding transcripts. Small RNAs, such as snoRNAs, were included in the analysis but constitute an exceedingly small portion of the overall noncoding DE transcripts identified. The reason for their underrepresentation is likely because small RNAs were not selected for in the initial RNA isolation process or in the polyA tail selection step of the library preparation process. Therefore, almost the entirety of the noncoding transcripts identified are lncRNAs, but this was by study design.

During RH infection, we identified 155 noncoding transcripts that were of higher abundance, and 94 transcripts that were of lower abundance (Fig. 4b). In comparison, 70 noncoding transcripts were of higher abundance and 13 were of lower abundance during PTG infection. When comparing RH to PTG infection, 22 noncoding transcripts were of higher abundance and 11 were of lower abundance. These 33 lncRNAs (Fig. 4d) are prime candidates to determine the role of lncRNA in parasite strain specific responses during infection. 31 noncoding transcripts were shared between RH and PTG infection (Fig. 4c and e). These 31 transcripts are the most likely candidates to be important for infection since they are similarly regulated in a strain-independent manner. A full list of noncoding DE transcripts for the three comparisons in *MyD88*^*+/+*^ BMDM (RH vs uninfected, PTG vs uninfected, and RH vs PTG) can be found in Additional file 10. Using qRT-PCR, differential expression of 4 lncRNAs (mir17hg, D43Rik, Loc105, and Gm19434) strongly validated the RNA-seq results (Additional file 11).

While at least three lncRNAs (Ftx, Snhg5 and Snhg15) have known functions, most of the DE long noncoding transcripts we identified have unknown function. However, many lncRNAs are associated with immune-related protein coding genes. Ftx, which is more abundant during RH infection, is a well-studied lncRNA with roles in cancer and X-chromosome inactivation [41, 42]. Two lncRNAs more highly abundant during RH infection, Snhg15 and Snhg5, are host genes for snoRNA. With roles in cancer, they appear to function as molecular sponges for microRNAs [43–46]. The conserved mir17hg lncRNA is a host gene for the mir17 microRNA cluster and is more abundant during both RH and PTG infection. Mir17 microRNAs are known to have a role in regulating apoptosis during *T. gondii* infection [29, 47, 48]. Interestingly, two Siva1 intronic lncRNAs (Siva1–203 and Siva1–205) were more abundant during RH infection, but the Siva1 protein-coding gene, an apoptosis-inducing factor, was not a DE. Similarly, three Nfkb1 intronic lncRNAs (Nfkb1–208, Nfkb1–210, and Nfkb1–202) were more abundant during RH infection but not the Nfkb1 protein-coding gene itself. While possibly an artifact, the differential abundance of these intronic sequences from their protein-coding counterpoints possibly suggests a function of these RNA species separate from the protein-coding gene. STAT1–210 (nonsense mediated decay) and STAT5a-207 (processed transcript) are differentially expressed in RH versus PTG and RH versus uninfected but the protein-coding transcript for these immune genes were not regulated. Several immune-related protein-coding genes (Socs2, ifi44, ifi203, ifi213) were co-regulated with overlapping lncRNAs (Socs2–209, Ifi44–203, ifi203–207, and ifi213–203).

### Sequencing of MyD88 KO mouse Transcriptome reveals few gene expression differences between MyD88^+/+^ and MyD88^−/−^ BMDM during *T. gondi* infection

**Fig. 5:**
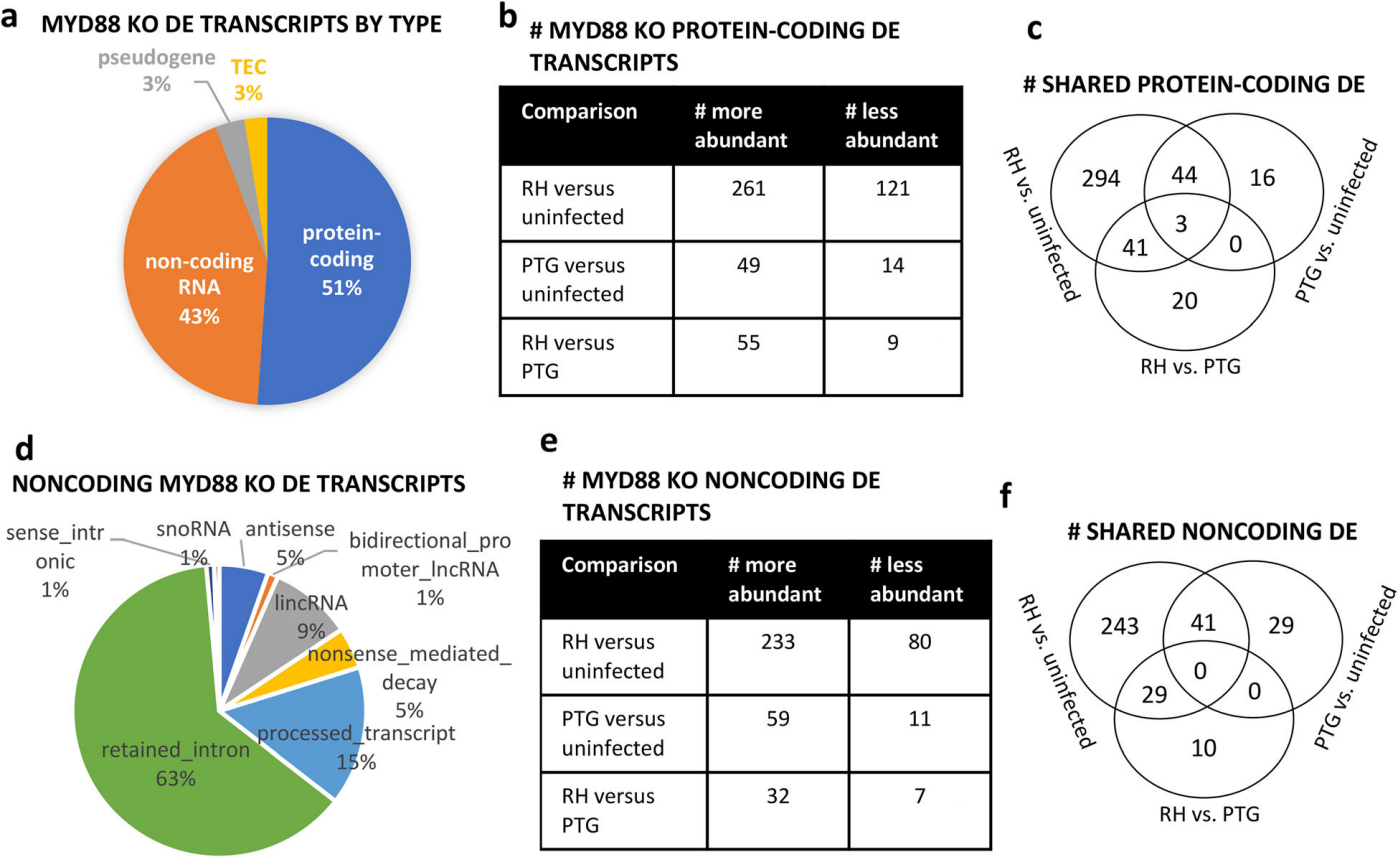
MyD88 KO BMDM have similar gene expression signatures as wild type BMDM. BMDM from MyD88 KO mice were infected with either the RH or PTG strain, and 6 h later RNA was isolated for sequencing. Differentially expressed mouse transcripts were identified based on statistical significance (PPDE greater than 0.95) and a fold change of greater or less than 2. **a** Classification of differentially expressed MyD88 KO transcripts as either protein-coding, non-coding, pseudogene, or TEC (To be Experimentally Confirmed). **b** Total number of protein-coding transcripts of higher or lower abundance during infection. **c** Venn diagrams of differentially expressed protein-coding transcripts. **d** Classification of differentially expressed MyD88 KO non-coding transcripts by type. **e** Total number of non-coding transcripts of higher or lower abundance during infection. **f** Venn diagrams of regulated non-coding transcripts revealing shared and unique expression patterns between infection strains. Experiments were performed in at least triplicate with BMDM from separate mice

The breakdown of all DE transcripts by type was remarkably similar for infection of wild type and MyD88 KO mice (Fig. 3a and Fig. 5a). This was also true for the noncoding transcripts more specifically (Figs. 4a and Fig. 5d). In general, the overall number of protein-coding and noncoding DE transcripts for RH and PTG infection was similar for wild type and MyD88 KO BMDM (Figs. 3b, 4b, 5b, and e). Likewise, the number of DE transcripts shared between RH and PTG was similar for wild type and MyD88 KO macrophages (Figs. 3c, 4c, 5c, and f). A full list of the protein-coding and noncoding DE transcripts for MyD88 KO macrophages is provided in Additional file 6 and Additional file 10.

**Fig. 6:**
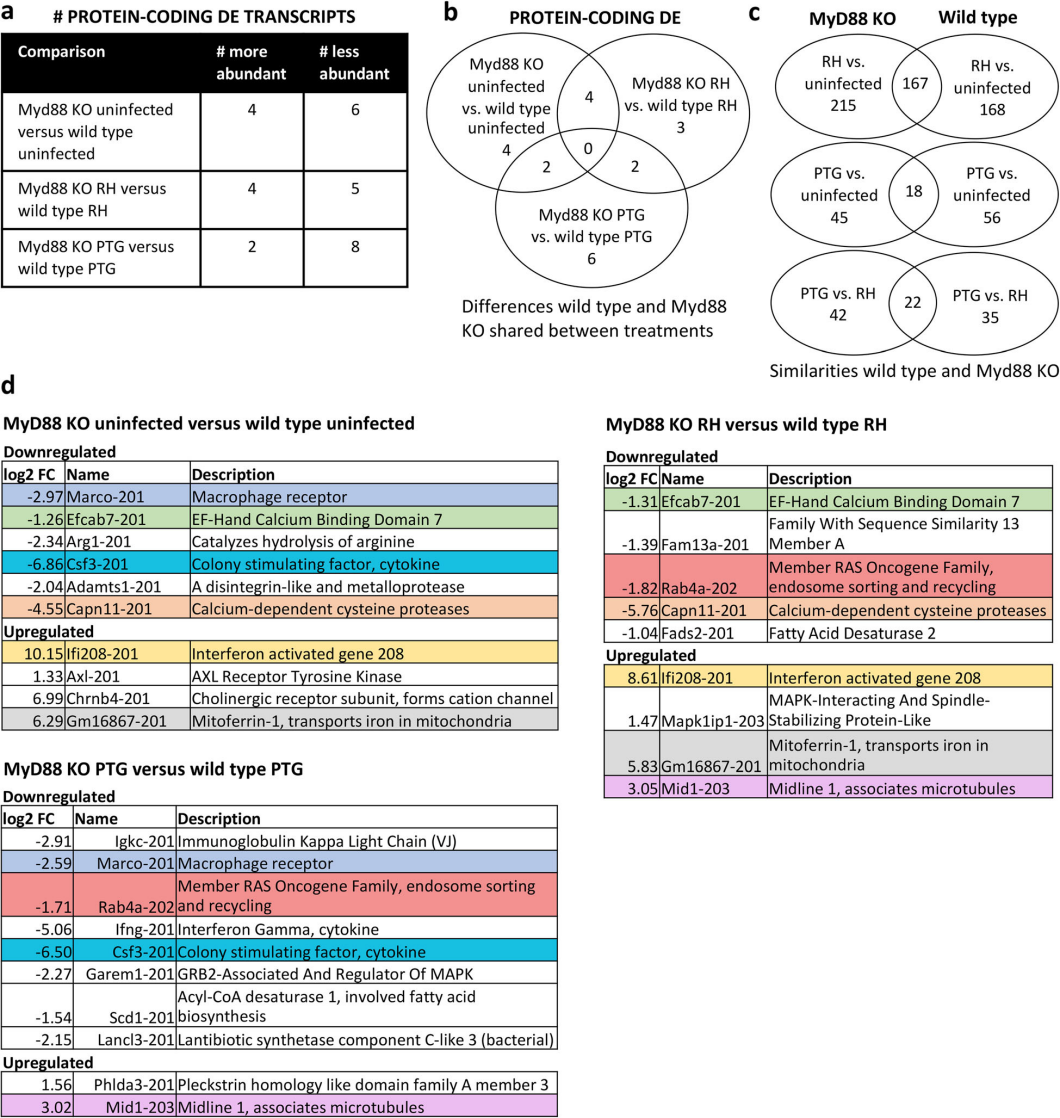
A minimal number of protein-coding genes were differentially expressed between wild type and MyD88 KO BMDM. Macrophages from wild type and MyD88 KO mice were infected with either the RH or PTG strain, and 6 h later RNA was isolated for sequencing. Differentially expressed mouse protein-coding transcripts between wild type and MyD88 KO BMDM were identified based on statistical significance (PPDE greater than 0.95) and a fold change of greater or less than 2. **a** Total number of protein-coding transcripts of higher or lower abundance between wild type and MyD88 KO mice for uninfected, RH-infected, and PTG-infected samples. **b** Venn diagrams of the gene expression differences between wild type and MyD88 KO mice showing shared expression changes for uninfected, RH-infected, and PTG-infected samples. **c** Venn diagrams revealing similarities between wild type and MyD88 KO gene expression changes for RH vs. uninfected, PTG vs. uninfected, and PTG vs. RH. **d** List of all differentially expressed protein-coding transcripts between wild type and MyD88 KO mice. Coloring indicates those transcripts shared between comparisons

**Fig. 7:**
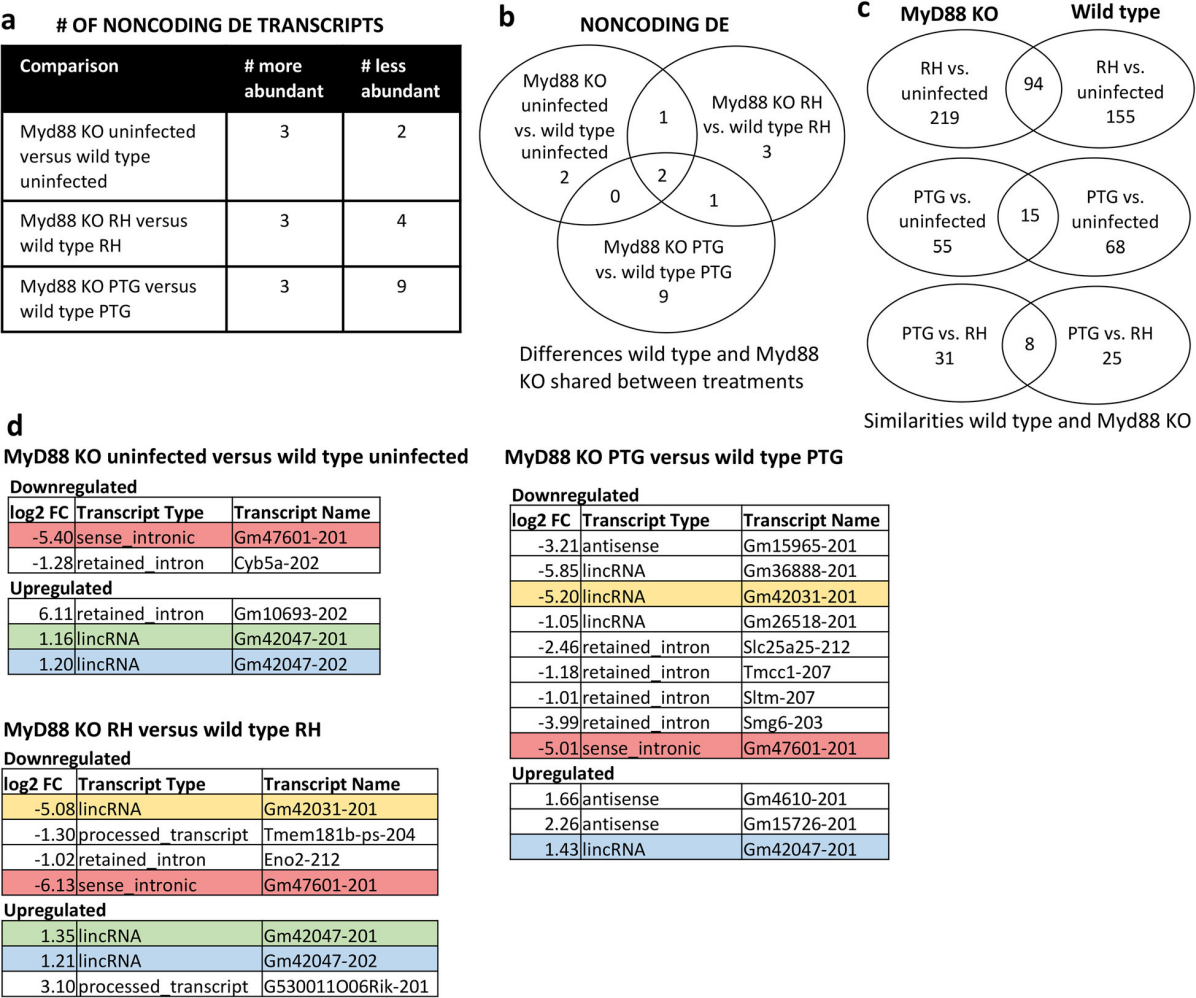
Only a few non-coding genes were differentially expressed between wild type and MyD88 KO BMDM. Cells from wild type and MyD88 KO mice were infected with *Toxoplasma* tachyzoites, and RNA was isolated for sequencing 6 h later. Differentially expressed mouse non-coding transcripts between wild type and MyD88 KO BMDM were identified based on statistical significance (PPDE greater than 0.95) and a fold change of greater or less than 2. **a** Total number of non-coding transcripts of higher or lower abundance between wild type and MyD88 KO macrophages for uninfected, RH-infected, and PTG-infected samples. **b** Venn diagrams of the non-coding gene expression differences between wild type and MyD88 KO cells revealing shared expression changes for uninfected, RH-infected, and PTG-infected samples. **c** Venn diagrams indicating similarities between wild type and MyD88 KO non-coding gene expression changes for RH vs. uninfected, PTG vs. uninfected, and PTG vs. RH. **d** List of all differentially expressed non-coding transcripts between wild type and MyD88 KO BMDM. Colored transcripts indicate those shared by different comparisons

We next focused on direct comparisons between MyD88^+/+^ and MyD88^−/−^ BMDM responses regarding protein-coding RNA (Fig. 6) and noncoding (Fig. 7) profiles. Only a few protein-coding and noncoding transcripts were differentially expressed between wild type and MyD88 KO BMDM (Figs. 6a and 7a). 10 protein-coding and 5 noncoding transcripts were differentially expressed between uninfected wild type and MyD88 KO macrophages. Similarly, 9 protein-coding and 7 noncoding transcripts were differentially expressed between RH infected wild type and MyD88 KO macrophages. Only 10 protein-coding and 12 noncoding transcripts were differentially expressed between PTG infected MyD88^+/+^ and MyD88^−/−^ BMDM. The degree of overlap between these three comparison groups is shown in Fig. 6b (coding transcripts) and Fig. 7b (noncoding transcripts).

In general, there was substantial overlap between the DE transcripts for wild type and MyD88 KO macrophages ranging from ~ 20–50% depending on the infection treatment (coding transcripts, Fig. 6c; noncoding transcripts, Fig. 7c). Functional analysis of the similarities between RH-infected wild type and MyD88 KO macrophages revealed enrichment of genes for cell cycle, DNA recombination, DNA replication, DNA repair, and cell migration (Additional file 7). Interestingly, immune genes were not functionally enriched in both MyD88^+/+^ and MyD88^−/−^ macrophages, despite differential expression of several immune genes such as ccl17, arg1, socs2, and ccl24 during RH infection of wild type and MyD88 KO cells. Regarding lncRNAs, greater abundance of mir17hg was common to both wild type and MyD88 KO infection by RH and PTG. Siva1–205 and Nfkb1–210 lncRNAs were of higher abundance during RH infection of wild type and Myd88 KO macrophages.

Several immune-related protein-coding genes were among the DE genes when comparing MyD88^+/+^ and MyD88^−/−^ macrophages, and a subset of these are known to be relevant in the response to *Toxoplasma*. Transcripts encoding the macrophage receptor Marco and the cytokine Csf3 were of lower abundance in the MyD88 KO BMDM relative to wild type cells for both uninfected and PTG treatment (Fig. 6d). Transcripts for Ifng (encoding IFN-γ) were less abundant in MyD88^−/−^ cells during PTG infection. IFN-γ is well known to be essential for survival of mice to acute *T. gondii* infection [8]. Transcripts for Arg1 (encoding arginase that catalyzes arginine hydrolysis) were less abundant in uninfected MyD88 KO macrophages. *Toxoplasma* is an arginine auxotroph and upregulation of Arg1 in wild type cells is thought to be a host defense mechanism to reduce levels of arginine [35, 49]. Ifi208 (interferon activated gene 208) is more abundant in MyD88 KO BMDM compared to wild type during RH infection.

Less is known regarding the differentially expressed noncoding RNAs identified. However, some results stand out. Two lncRNAs, Gm42047–201 and Gm47601–201, were differentially expressed among all three conditions (MyD88 KO uninfected vs. wild type uninfected, MyD88 KO RH versus wild type RH, and MyD88 KO PTG versus wild type PTG) suggesting these two lncRNAs may be important in the difference between the two macrophage strains (Fig. 7d). 5 of the 18 noncoding transcripts (28%) are long intergenic noncoding RNAs meaning they are not near or overlapping any protein-coding genes. This is of practical interest because intergenic lncRNAs are more amenable to genetic knock down, making them easier targets to study. One lncRNA, Sltm-207, is intronic to the protein-coding gene SLTM and is less abundant in MyD88 KO compared to wild type during PTG infection. When overexpressed, SLTM acts a general inhibitor of transcription that eventually leads to apoptosis [50].

### Sequencing of the *Toxoplasma* Transcriptome revealed no differences in gene expression between infection of wild type and MyD88 KO macrophages

*Toxoplasma* reads were mapped to the ToxoDB ME49 strain and differentially expressed genes were identified (*p*-value equal or less than 0.05 and fold change of 2 or greater). Because both Type I and Type II strains were mapped to ME49 (Type II), genetic differences between the strains could result in issues mapping to specific genes. While the overall mapping rate to RH was higher than for PTG, discrepancies for specific genes could exist. Additionally, the number of *T. gondii* reads obtained for many of the samples was below our projected target of 10 M reads per sample (actual range was between 1.2 M and 10 M reads per sample). This limited our ability to detect lowly expressed genes and could result in detecting fewer DE genes overall. Because we also did not analyze a non-infection control for *T. gondii* sequences (which is difficult to do), we conducted fewer comparisons than for the mouse transcriptome data. We directly compared RH and PTG infection for both wild type and MyD88 KO macrophages. We also compared wild type versus MyD88 KO, for RH and PTG, respectively.

**Fig. 8:**
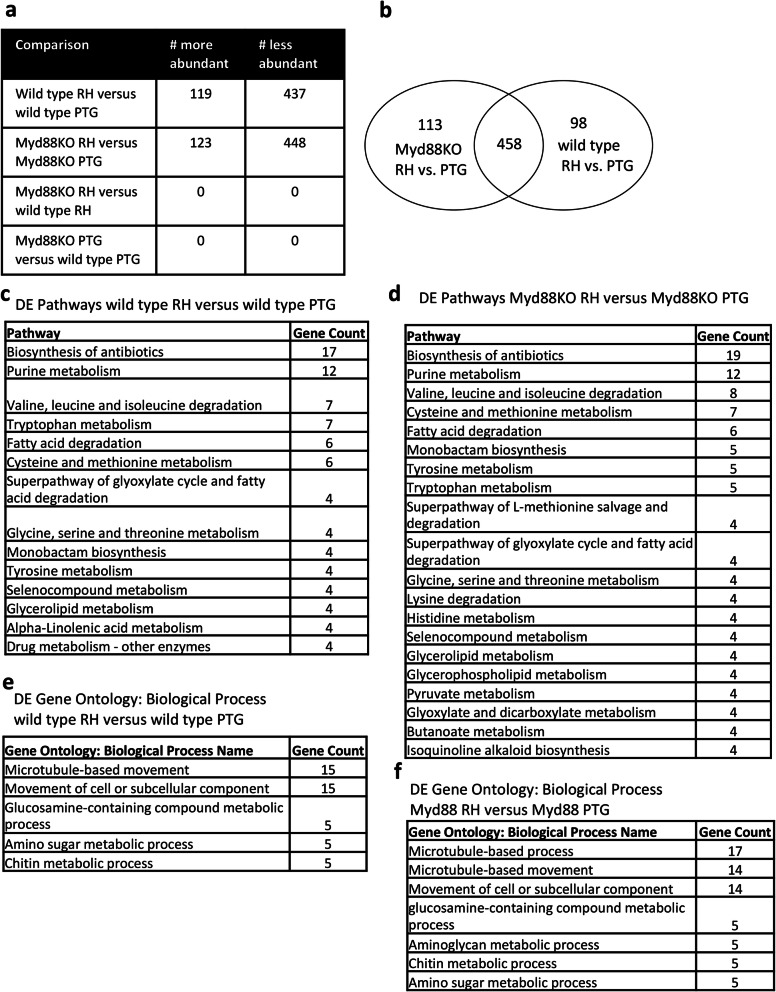
Greater than 550 *Toxoplasma gondii* genes were differentially expressed between the highly virulent RH strain and the less virulent PTG strain, but none were significantly different between wild type mice and MyD88 KO mice. BMDM from wild type and MyD88 KO mice were infected with RH or PTG tachyzoites, and 6 h later RNA was isolated for sequencing. Differentially expressed *T. gondii* genes were identified based on statistical significance (PPDE greater than 0.95) and a fold change of greater or less than 2. **a** Total number of *T. gondii* genes of higher or lower abundance for each comparison. **b** Venn diagrams showing shared expression changes between wild type and MyD88 KO samples for RH vs. PTG. **c** Pathways enriched between RH and PTG infection of wild type macrophages **d** Pathways enriched between RH and PTG infection of MyD88 KO BMDM. **e** Biological process enrichment between RH and PTG infection of wild type macrophages. **f** Biological process enrichment between RH and PTG infection of MyD88 KO cells

For both wild type and MyD88 KO, approximately 120 parasite genes were more abundant in RH infection compared to PTG infection (Fig. 8a). Unexpectedly, a larger number of genes (approximately 440) were less abundant in RH compared to PTG parasites (Fig. 8a). The opposite was true when comparing mouse differential gene expression between RH and PTG (Fig. 3b). More than half (approximately 55%) of the parasite DE transcripts are hypothetical proteins with no known function or homology to other proteins with known functions (Additional file 12). Surprisingly, no differentially expressed *Toxoplasma* genes were identified when comparing MyD88 KO RH versus wild type RH and MyD88 KO PTG versus wild type PTG host strains (Fig. 8a). This indicates that *Toxoplasma* behaves the same regardless of the host macrophage MyD88 genotype. Most (approximately 80%) of the differentially expressed genes between RH and PTG were shared between wild type and MyD88 KO strains (Fig. 8b and Additional File 12). This high degree of similarity is consistent with the absence of significantly differentially expressed genes between *T. gondii* infection of wild type and MyD88 KO strains.

Pathway analysis identified metabolism of purines and several amino acids as different between RH and PTG infection (Fig. 8c-f). Overall, these transcripts were largely less abundant during RH infection compared to PTG. Gene ontology analysis identified microtubule-based movement as less abundant during RH infection compared to PTG infection (Fig. 8e and f). Several motor proteins (myosin, dynein, intraflagellar transport protein, and kinesin) were less abundant in RH versus PTG in both wild type and MyD88 KO (Additional file 12). However, two dynein proteins were more abundant in RH versus PTG for both wild type and MyD88 KO. *T. gondii* is known to use motor proteins for invasion into the host and for replication [51, 52]. Several SAG-related sequences (which encode a major family of tachyzoite surface proteins) were more abundant in RH versus PTG but a subset was also less abundant (Additional file 12). Many secretory proteins (ROP38, GRA11, ROP28, ROP2A, ROP23, ROP46, MIC17B, and ROP4) were less abundant in RH versus PTG in both wild type and MyD88 KO. GRA15 was more abundant in RH versus PTG but interestingly only during infection of MyD88^−/−^ BMDM. The rhoptry protein ROP8 was less abundant in RH versus PTG only in MyD88 KO host cells. Oocyt wall protein (OWP1) was less abundant in RH versus PTG in both wild type and MyD88^−/−^ macrophages. Transcripts for the bradyzoite antigen BAG1 were less abundant during RH infection compared to PTG infection in both wild type and MyD88 KO. This is consistent with the RH strain being unable to form cysts during infection. We validated the lower abundance of Bag1 in RH versus PTG using qRT-PCR (Additional File 13).

**Fig. 9:**
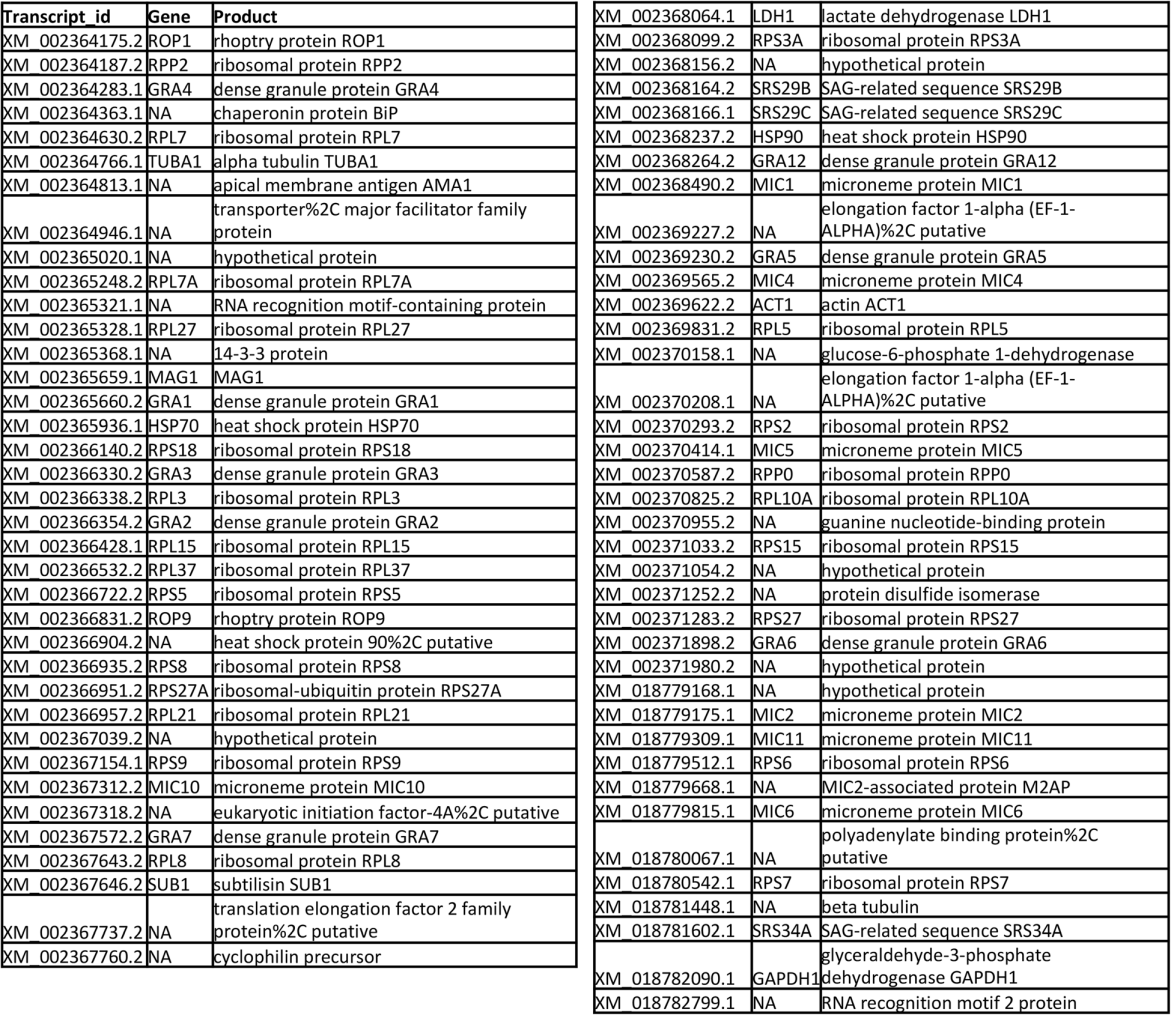
Rhoptry, microneme, and dense granule genes are among the most abundant *Toxoplasma* transcripts. Using normalized expression values, the top 100 expressed genes were compared between the four samples (RH infecting wild type, RH infecting MyD88 KO, PTG infecting wild type, and PTG infecting MyD88 KO). 75 out of 100 were shared between all four samples and those shared are listed here

Independently of the DE analysis, we examined which *Toxoplasma* genes were most highly expressed during infection. Using normalized expression values, we compared the top 100 expressed genes between all four samples (RH infection of wild type and MyD88^−/−^ cells; PTG infection of wild type and MyD88^−/−^ BMDM). 75 out of 100 most expressed genes were shared between all four samples and these are listed in Fig. 9. The most highly expressed gene was that encoding rhoptry protein ROP1, a secretory molecule whose function is unclear. Many other *T. gondii* secreted proteins were represented, including dense granule proteins (8 in total), rhoptry proteins (2 in total), and microneme proteins (7 in total). Together, they comprise 22.7% of the most highly expressed genes. 23 out of 75 genes (30.7%) were ribosomal proteins. Only six out of the 75 genes (8%) were hypothetical, substantially less than the overall percentage of hypothetical proteins.

## Discussion

In this study, we undertook a global analysis of the coding and noncoding mouse transcriptome during Type I and Type II *T. gondii* infection of wild type and MyD88 KO BMDMs. Numerous previous studies have examined the host protein-coding response to infection using RNA-sequencing in various parasite strains, cells/organs, and host species, including bone-marrow derived macrophages and bone-marrow derived dendritic cells [49, 53–59]. While not the primary focus of our study, we identified many similarities to previous studies in the wild type mouse protein-coding response, including dysregulation of immune and metabolic genes. One of our more interesting findings is that significantly more host genes were differentially expressed during Type I rather than Type II infection. Our studies were conducted on cells infected for 6 h, prior to the first parasite mitotic event. Thus, strain-specific differences in replication do not account for the effect. This indicates that Type II infection is overall more silent than Type I infection, a finding that is consistent with in vivo evidence in mice that Type II parasites overall cause less severe disease than Type I strains [60, 61]. Of interest is to consider the transcriptomic changes during RH and PTG infection in light of the polymorphic effector molecules expressed by these strains. Type I parasite strains such as RH express an active form of ROP16. This rhoptry protein is a host-directed kinase that activates STAT3, 5 and 6 signaling pathways that down-regulate several immune response genes [35, 62]. In our results, we found that many immune genes were both more and less abundant during *T. gondii* infection, clearly indicating that other factors, in addition to ROP16, are relevant to macrophage infection. Conversely, PTG, a type II strain, expresses GRA15, a secreted dense granule protein that activates several proinflammatory genes through an NFκB pathway [63]. This is perhaps in contradiction to our findings, where most host immune genes were only weakly affected by PTG infection. The influence of these parasite effector molecules on the transcriptional responses reported here await further investigation.

Another interesting finding was the higher abundance of cell migration genes in both the RH versus uninfected and RH versus PTG comparisons. This is consistent with data indicating that *T. gondii* enhances the motility of dendritic cells, a phenomenon hypothesized to facilitate dissemination throughout the host [18, 64]. Our results suggest that this phenomenon extends beyond dendritic cells to macrophages as well.

In this study, the parasites were centrifuged and washed prior to infecting BMDM, but parasite preparations likely contain small amounts of fibroblast debris. We cannot rule out that the presence of some fibroblast debris could possibly have their own effects on gene expression. If so, this would most likely apply to mouse genes differentially expressed during both RH and PTG infection and less likely to affect only one strain, as the two strains were treated identically with regard to centrifugation and washing. The possibility that fibroblast factors rather than parasites per se mediate some of the effects seen is minimized by the fact that the fibroblasts are human in origin whereas the macrophages used for infection are mouse derived.

While many studies have assessed the protein-coding transcriptomic response to *T. gondii* infection, very few have looked at the noncoding RNA response to infection. Two previous studies examined expression of lncRNAs in human cells during *T. gondii* infection. One, using microarrays, found that at least 1000 human lncRNAs were differentially expressed during Type II infection of HFF cells [65]. Another used qRT-PCR to assess a panel of lncRNAs with known immune regulatory activity and found 31 lncRNAs were differentially expressed during *T. gondii* infection of retinal müller glial cells [66]. Despite the low degree of sequence conservation between human and mouse lncRNAs, two lncRNAs, Mir17hg and Snhg15, differentially expressed in the Rochet et al. study were also differentially expressed in this study.

We previously looked at the expression of lncRNAs in mouse cells during *T. gondii* infection using microarrays [67]. In that study, we found 1522 lncRNAs differentially expressed during infection with the RH strain and 528 with the PTG strain. Here, we found 249 noncoding transcripts as differentially expressed during Type I infection and 83 noncoding transcripts as differentially expressed during Type II infection of wild type mouse macrophages. While we identified a smaller number of lncRNAs with RNA-sequencing than with microarrays, we found significant overlap including Mir17hg, Ftx, and Socs2–209. While difficult to directly compare, we identified approximately 55 lncRNAs shared between the two datasets based on gene names. We identified a larger number (155) of shared DE transcripts for mRNAs, possibly due to either more consistent naming or higher expression of protein-coding genes.

In this study, we identified 33 lncRNAs that were differentially expressed between RH and PTG. These would be important to pursue for their role in strain specific differences between Type I and Type II *T. gondii.* Additionally, 31 noncoding transcripts were differentially expressed in both RH and PTG infection. These 31 transcripts are perhaps most likely to be essential for infection as they are regulated in both strains. This study, combined with previous studies, provides a sense of the vast landscape of lncRNAs regulated during *T. gondii* infection whose functions we do not yet understand. Given the large number of lncRNAs identified in this study, as well as the emerging role of lncRNAs in regulating the immune response [30, 68], we believe this is an important avenue to investigate further in order to fully understand the immune response to *T. gondii* and other intracellular pathogens impacting human health. Functional studies employing approaches such as knockout or overexpression of lncRNAs is a necessary next step.

The present study is the first to examine the transcriptional response of MyD88 deficient mouse cells during *T. gondii* infection. The MyD88 protein plays an important role in the early innate immune response in mice to *T. gondii* infection [69]. The effect of MyD88 deletion on expression of downstream immune genes in infected cells was previously unknown. Additionally, this is an important avenue of research because the human immune response to *Toxoplasma* is not thought to depend on the TLR/MyD88 axis, and there is much interest in determining other avenues of immune recognition in humans and mice. Perhaps surprisingly, only a few genes were differentially expressed between wild type and MyD88 KO mice. This suggests that MyD88 has only minor effects on cellular transcriptional activity, at least within the time frame of our study. Several immune genes and two meiotic genes were among the differences. Two lncRNAs (Gm42047–201 and Gm47601–201) were differentially expressed among all three conditions (RH, PTG, and noninfected), suggesting that these two lncRNAs may be especially important in the MyD88-dependent immune response. Further investigation of these protein-coding genes and non-coding transcripts in the context of the MyD88 KO immune response is important.

While not the primary focus of our study, we also examined parasite gene expression for RH and PTG infection of wild type and MyD88 KO macrophages. More than 550 genes were differentially expressed between Type I and Type II *T. gondii*. In both the RNA-sequencing dataset and qRT-PCR validation, the Bag1 gene was less abundant in the RH strain versus the PTG strain. This result is unsurprising as Bag1 expression is specific to the bradyzoite stage of the parasite, a life cycle stage which the RH strain is incapable of achieving. Several microtubule and motor proteins (myosin, dynein, intraflagellar transport protein, and kinesin) were less abundant in RH versus PTG in both wild type and MyD88 KO. This result is surprising given the much faster growth rate of the RH strain, however, two dynein proteins were more abundant during RH infection. It is interesting that the most highly expressed *Toxoplasma* gene across samples was the rhoptry protein ROP1, a secretory molecule whose function is unknown. Determining the function of this protein could be interesting and important. Previous studies have used RNA-sequencing to examine *T. gondii* gene expression patterns [54–56, 58, 70]. In 2014, Pittman et al. compared acute and chronic Type II *T. gondii* infection of mouse brains. While difficult to directly compare the two studies, we also found that metabolic, biosynthetic, and translation genes were among the most highly expressed *T. gondii* genes regardless of strain type. In 2016, Zhou et al. examined RH gene expression during infection of porcine PK-15 cells. They found that metabolic genes were largely less abundant in intracellular RH tachyzoites compared to egressed parasites. We also found that many metabolic genes were less abundant in RH when compared to PTG infection. One of our more interesting findings is that no *T. gondii* genes were differentially expressed between wild type and MyD88 KO macrophages, suggesting that *T. gondii* behaves the same regardless of which host strain it infects. Nevertheless, it is possible that at later infection time points differences in the parasite transcriptome in wild type and KO cells would emerge.

## Conclusions

While this study covered numerous disparate aspects of early intracellular infection, we were largely focused on two facets: (1) identifying long noncoding RNAs differentially expressed during *T. gondii* infection and (2) examining the transcriptional response of MyD88 KO cells. Host lncRNAs represent a largely unexplored layer in the immune response to *T. gondii*. The MyD88-independent transcriptional response may also be important in unraveling other mechanisms of immune regulation in the response to *T. gondii* in both humans and mice.

## Methods

### Mouse strains and generation of BMDM

C57BL/6 mice (The Jackson Laboratory) were used as the wild type strain in this study. The MyD88 KO mice contain a deletion of exon 3 (The Jackson Laboratory, B6.129P2(SJL)-*Myd88tm1.1Defr*/J). Animals were housed (3–5 per cage) and cared for according to the Guide for the Care and Use of Laboratory Animals (8th edition) under an IACUC approved protocol (19–200,854-MC). Mice were housed in 3 single-sided ventilated cage racks (RAIR HD Super Mouse 1800 Interchangeable Micro Isolator Unit 9 tier). The mice were housed in a specific pathogen-free facility under a 12:12 light/dark cycle with controlled temperature (22–24 °C) and humidity (50–60%). Mice were given free access to autoclaved standard mouse chow and water and were checked every 24 h for general health. Animals were rested for 1 week prior to generating BMDM. We used a total of 8 wild type mice and 8 MyD88 KO mice in this study. All mice were females between the ages of 6 and 8 weeks (17–20 g). The mice were euthanized using CO_2_ asphyxiation, as recommended by the American Veterinary Medical Association. Mice were euthanized in the morning in a separate room from caged mice. Bone marrow from a single mouse was used for each biological replicate for each mouse strain, with four biological replicates from four separate mice shown (4 replicates for uninfected mice samples and 3 replicates for infected samples). Femur and tibia were used as a source of bone marrow cells. Macrophages were generated from single cell suspensions of bone marrow cells by 5- or 6-day culture in L929-containing supernatants, as previously described [71]. One day prior to infection, BMDM were harvested, counted, and plated in 12-well tissue culture plates in human fibroblast medium (HFM) at a concentration of 1 × 10^6^ cells per well. HFM consists of DMEM (Life Technologies) supplemented with 1% heat-inactivated bovine growth serum (Thermo Fisher Scientific), 100 U/mL penicillin (Life Technologies), and 0.1 mg/mL streptomycin (Life Technologies).

### Parasite strains and infections

Wild type *Toxoplasma* strains RH (Type I) and PTG (Type II) were used in this study. Tachyzoites of both strains were maintained by approximately twice-weekly passage on human foreskin fibroblast monolayers in HFM. Flasks of fully lysed fibroblasts were utilized for infections. If fibroblasts were only partially lysed, they were passed twice through a 27 Ga needle to release the parasites. All parasites were centrifuged and washed prior to infection to remove soluble material. Infections were accomplished by the addition of tachyzoites to mouse BMDM (4:1 or 5:1 ratio of parasites to cells) on 12-well plates (Falcon, non-tissue culture treated). Plates were briefly centrifuged (3 min, 200×g) to initiate contact between tachyzoites and macrophages. Cultures were incubated 6 h (37 °C, 5% CO2), then the cells were harvested for RNA extraction. Percent infection was calculated using an Olympus BX51 immunofluorescence microscope. DAPI was used to stain the nucleus. *Toxoplasma*-specific polyclonal antibody conjugated to FITC (ThermoFisher Scientific) was used to stain the parasites. Percent infection of at least 70% was deemed acceptable.

### RNA-seq sample preparation and Illumina NextSeq 500 sequencing

Total RNA was prepared from BMDM by RNeasy Mini Kit purification (Qiagen). The samples were subjected to Turbo DNase treatment (Life Technologies). Total RNA from each sample was quantified using a Qubit 3.0 Fluorometer. At least 1.5 μg per sample was submitted to the CETI Molecular Biology Core at the University of New Mexico, and RNA integrity was assessed using an Agilent 2100 Bioanalyzer. All samples had RIN values > 9.6, indicating the RNA was high quality. Libraries were prepared using the KAPA mRNA HyperPrep Kit with PolyA selection. In brief, mRNA was captured using magnetic oligo-dT beads. RNA was fragmented using heat and magnesium, and first strand was synthesized by random priming. Second strand synthesis and A-tailing were combined into one step, which involves converting the cDNA:RNA hybrid to double-stranded cDNA while incorporating dUTP into the second cDNA stand and adding dAMP to the 3′ ends of the double-stranded cDNA fragments. Next, dsDNA adapters with 3′-dTMP were ligated to the A-tailed library fragments, and the library fragments with adapters at both ends were amplified using PCR. The strand marked with dUTP was not amplified, allowing strand-specific sequencing. Library quality was assessed using a Qubit and a Bioanalyzer. qRT-PCR of adapters was performed to determine the amount of library in each sample. Libraries were sequenced using an Illumina NextSeq 500 Sequencer generating paired-end, 75-bp read length sequences. 1 mid-throughput run (150 M theoretical maximum clusters) and 3 high-throughput runs (400 M theoretical maximum clusters) were performed. Figure 1 indicates the total number of reads obtained per sample.

### RNA-seq data analysis

Raw reads off sequencer were trimmed and filtered using Trimmomatic v0.36 (Bolger et al. 2014) with slide window of 4 nt, average score above 20 and minimum length of 36 nt. Filtered high quality reads were mapped to the annotated mouse genome release M21 from GenCode [72] and *T. gondii* ME49 (GenBank Assembly ID GCA_000006565.2) using RNA-Seq reads mapping tool Spliced Transcripts Alignment to a Reference (STAR) 2.5.3a [73]. Reads mapping were performed using STAR options: --genomeLoad LoadAndKeep --limitBAMsortRAM 50,000,000,000 --runMode alignReads --runThreadN 17 --outBAMsortingThreadN 7. No major changes were made to default settings other than to tune CPUs and memory. Gene expression levels were estimated using software featureCounts with default settings [74]. Differential gene expression (DE) analysis was performed using EBSeq v1.22.0 default settings [75]. A posterior probability of differential expression (PPDE) 0.95 or higher for EBSeq was set as cutoff for DE analysis. Workflow of read trimming and mapping was built using Unix shell commands with application GNU-Parallel [76] to perform jobs in parallel. Table summary and figure generation were performed with the R statistical computing environment [77] and Bioconductor [78], including the following packages: ggplot2 [79], reshape2 [80], and pheatmap v1.0.12 [81]. Heat maps were constructed using complete-linkage clustering using log2 fold change values or the z-score transformed FPKM values. PCA plots were constructed using SARTools v1.6 [82]. For mouse genes, functional enrichment analysis, including KEGG pathway analysis & Gene Ontology analysis, was performed using g:Profiler (g:Profiler version *e96_eg43_p13_563554d*) with g:SCS multiple testing correction method applying significance threshold of 0.05 [83]. For *T. gondii* genes, functional enrichment analysis was performed using g:Profiler (for gene ontology data) and ToxoDB Identify Metabolic Pathway tool (for KEGG pathway data) [84]. Venn diagrams, pie charts, and other data analysis was performed using Microsoft Excel software.

### Quantitative RT-PCR

Total RNA was prepared using a RNeasy Mini Kit (Qiagen), and the samples were subjected to Turbo DNase treatment (Life Technologies). RNA was converted to cDNA using the SuperScript IV VILO Master Mix (ThermoFisher Scientific). Quantitative PCR was performed on target genes and normalized to the expression of the housekeeping gene *Ppia* (mouse gene) or β-tubulin (*T. gondii* gene) using the SYBR green method (SsoAdvanced Universal SYBR Green Supermix, Bio-Rad) and the Bio-Rad CFX96 RT-PCR machine. Expression relative to uninfected control samples was calculated using the ∆∆Ct method. A control with no added reverse transcriptase was included for each sample. Primer sequences used in this study are Ppia (F-GCATGTGGTCTTTGGGAAGGTG and R-GGGTAAAATGCCCGCAAGTCAA), Mir17hg (F-GGTGGCCACTCTGTTAATGTGC and R-TAACTGCAGCTTCTCCAGACCC), D43Rik (F-TTCTCAATACAACGCCCCAGGT and R-AAAAGGGGGAAGGATTGGGGAG), LOC105 (F-GGCACACCCATGATAGGCTGTA and R-CCTCCATACCCAGCACTGTCAA), Gm19434 (F-TGACAGGGAAAAACAGAGCCCA and R-GTCACCCAGGGGAAAGCAATTG), β-tubulin (F-CGCCACGGCCGCTACCTGACT and R-TACGCGCCTTCCTCTGCACCC), and Bag1 (F-GACGTGGAGTTCGACAGCAAA and R-ATGGCTCCGTTGTCGACTTCT). Some of our uninfected samples were occasionally at the limit of detection of our qRT-PCR assay. In those cases, we used 35 cycles as the Cq (quantification cycle).

## Supplementary Information

**Additional file 1.** Principal component analysis (PCA) plots of mouse RNA-sequencing data reveal associations between samples: PCA plots of mouse transcripts for wild type (wt) BMDM comparisons.

**Additional file 2.** Principal component analysis (PCA) plots of mouse RNA-sequencing data reveal associations between samples: PCA plots of mouse transcripts for MyD88 KO (k88) BMDM comparisons.

**Additional file 3.** Principal component analysis (PCA) plots of mouse RNA-sequencing data reveal associations between samples: PCA plots of mouse transcripts for MyD88 KO BMDM versus wild type BMDM comparisons.

**Additional file 4 **Principal component analysis (PCA) plots of *T. gondii* RNA-sequencing data reveal associations between samples: PCA plots of *T. gondii* transcripts for MyD88 KO (k88) BMDM versus wild type (wt) BMDM comparisons.

**Additional file 5 **Principal component analysis (PCA) plots of *T. gondii* RNA-sequencing data reveal associations between samples: PCA plots of *T. gondii* transcripts for RH versus PTG comparisons.

**Additional file 6.** Complete list of differentially regulated mouse protein-coding genes in MyD88^+/+^ and MyD88^−/−^ BMDM. Each tab in the spreadsheet corresponds to a specific pairwise comparison (e.g., RH vs. uninfected). Transcript name, Gene IDs, raw values, normalized values, PPDE values, fold changes, and other information are included.

**Additional file 7.** Functional analysis of differentially expressed protein-coding genes. Each tab in the spreadsheet corresponds to a specific pairwise comparison (e.g., RH vs. uninfected). Results were obtained using g:Profiler. Gene Ontology: Biological Process and KEGG Pathway analysis results are included.

**Additional file 8.** Heatmaps displaying individual replicates of differentially expressed immune and cell cycle genes in wildtype mice. Heatmaps displaying z-score transformed FPKM values of individual replicates among functionally related genes.

**Additional file 9.** Heatmaps displaying individual replicates of differentially expressed growth, cell adhesion, and migration genes in wildtype mice. Heatmaps displaying z-score transformed FPKM values of individual replicates among functionally related genes.

**Additional file 10.** Complete list of differentially expressed mouse non-coding genes. Each tab in the spreadsheet corresponds to a specific pairwise comparison (e.g., RH vs. uninfected). Transcript name, Gene IDs, raw values, normalized values, PPDE values, fold changes, and other information are included.

**Additional file 11 **RNA-seq lncRNA data validation by qRT-PCR. RNA from mouse BMDM were collected 6 h after infection with either RH or PTG strains of *T. gondii*, and qRT-PCR was performed. Fold changes represent the comparison of infected samples to uninfected samples. (a) qRT-PCR fold change values for 4 lncRNAs. (b) RNA-seq fold change values for the same 4 lncRNAs for comparison purposes. Experiments were completed at least three times with BMDM from three separate mice.

**Additional file 12 **Complete list of differentially expressed *Toxoplasma gondii* genes. The first tab corresponds to wild type RH versus wild type PTG. The second tab corresponds to Myd88KO RH versus Myd88KO PTG. The third tab lists the differentially regulated *T. gondii* genes common to both comparisons. Transcript ID, raw values, normalized values, PPDE values, fold changes, and other information are included.

**Additional file 13 **RNA-seq *Toxoplasma gondii* data validation by qRT-PCR. RNA from mouse BMDM were collected 6 h after infection with either RH or PTG strains of *T. gondii*, and qRT-PCR was performed. Fold changes represent the comparison of RH infected samples to PTG infected samples. RNA-sequencing and qRT-PCR data are displayed in one graph. Experiments were completed at least three times with BMDM from three separate mice.

## Data Availability

The datasets generated and/or analysed during the current study are available in the NCBI Sequence Read Archive (SRA) repository, PRJNA695200 at https://www.ncbi.nlm.nih.gov/bioproject/695200. Reads were mapped to the annotated mouse genome release M21 from GenCode (GenBank assembly ID GCA_000001635.2) and *T. gondii* ME49 (GenBank Assembly ID GCA_000006565.2).

## Abbreviations

BMDM: Bone marrow-derived macrophages
Cq: quantification cycle
DE: Differentially expressed
GENCODE: Encyclopedia of DNA elements
HFM: Human fibroblast medium
KEGG: Kyoto encyclopedia of genes and genomes
KO: Knockout
lincRNA: long intergenic noncoding RNA
lncRNA: long noncoding RNA
Nt: Nucleotide
PCA: Principal component analysis
PPDE: Posterior probability of differential expression
qRT-PCR: quantitative reverse transcriptase polymerase chain reaction
RIN: RNA integrity
RNA-seq: RNA sequencing
snoRNA: small nucleolar RNA
TEC: To be experimentally confirmed
TLR: Toll-like receptor
