# Supplementary figures and images for: Transcriptomics analysis of Toxoplasma gondii-infected mouse macrophages reveals coding and noncoding signatures in the presence and absence of MyD88
Source: BMC Genomics. 2021 Feb 23;22:130. doi: 10.1186/s12864-021-07437-0 (PMC7903719; doi:10.1186/s12864-021-07437-0)

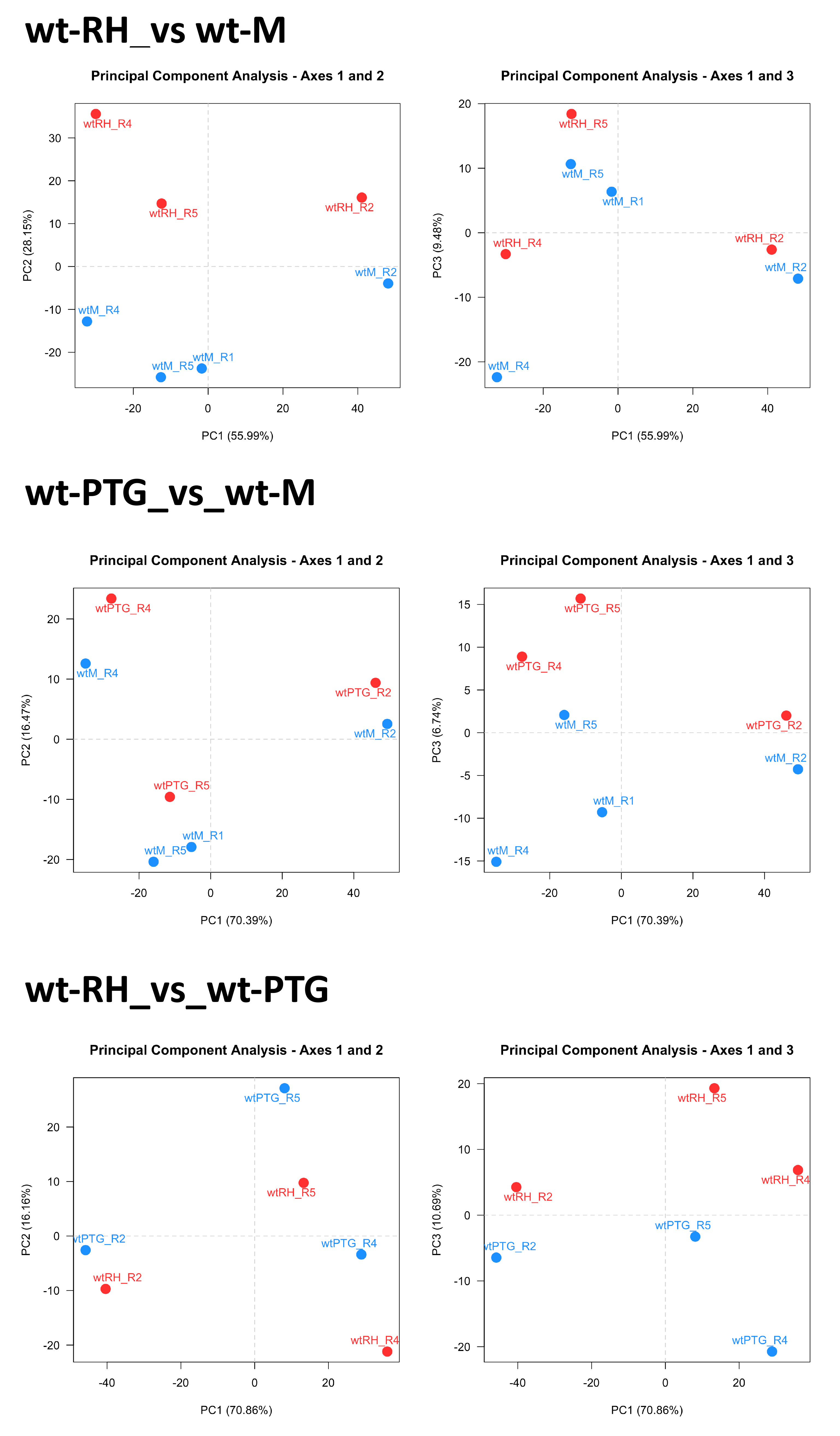

Supplement: Supplementary file 1 — Additional file 1. Principal component analysis (PCA) plots of mouse RNA-sequencing data reveal associations between samples: PCA plots of mouse transcripts for wild type (wt) BMDM comparisons. [file 12864_2021_7437_MOESM1_ESM.tif]

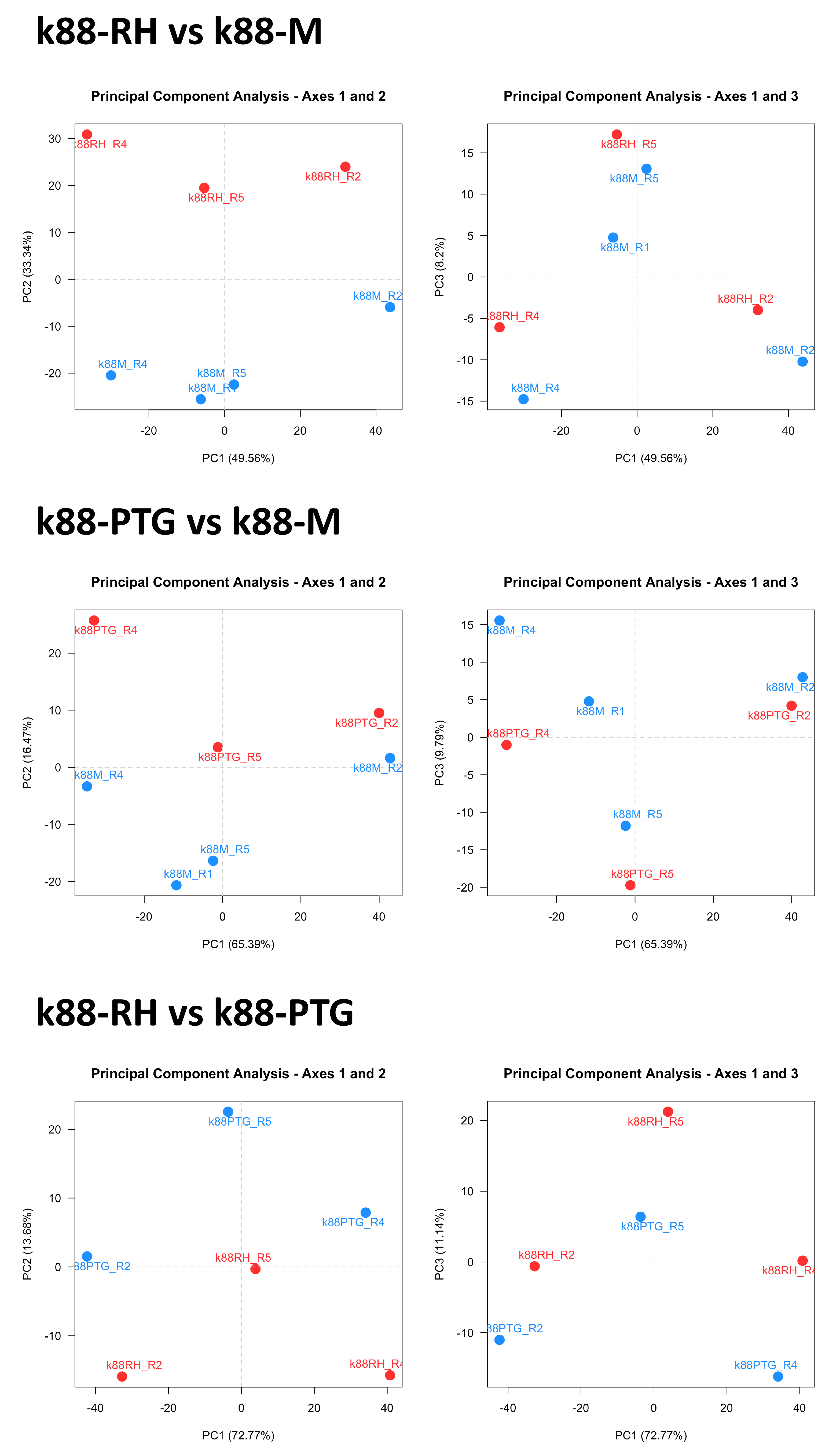

Supplement: Supplementary file 2 — Additional file 2. Principal component analysis (PCA) plots of mouse RNA-sequencing data reveal associations between samples: PCA plots of mouse transcripts for MyD88 KO (k88) BMDM comparisons. [file 12864_2021_7437_MOESM2_ESM.tif]

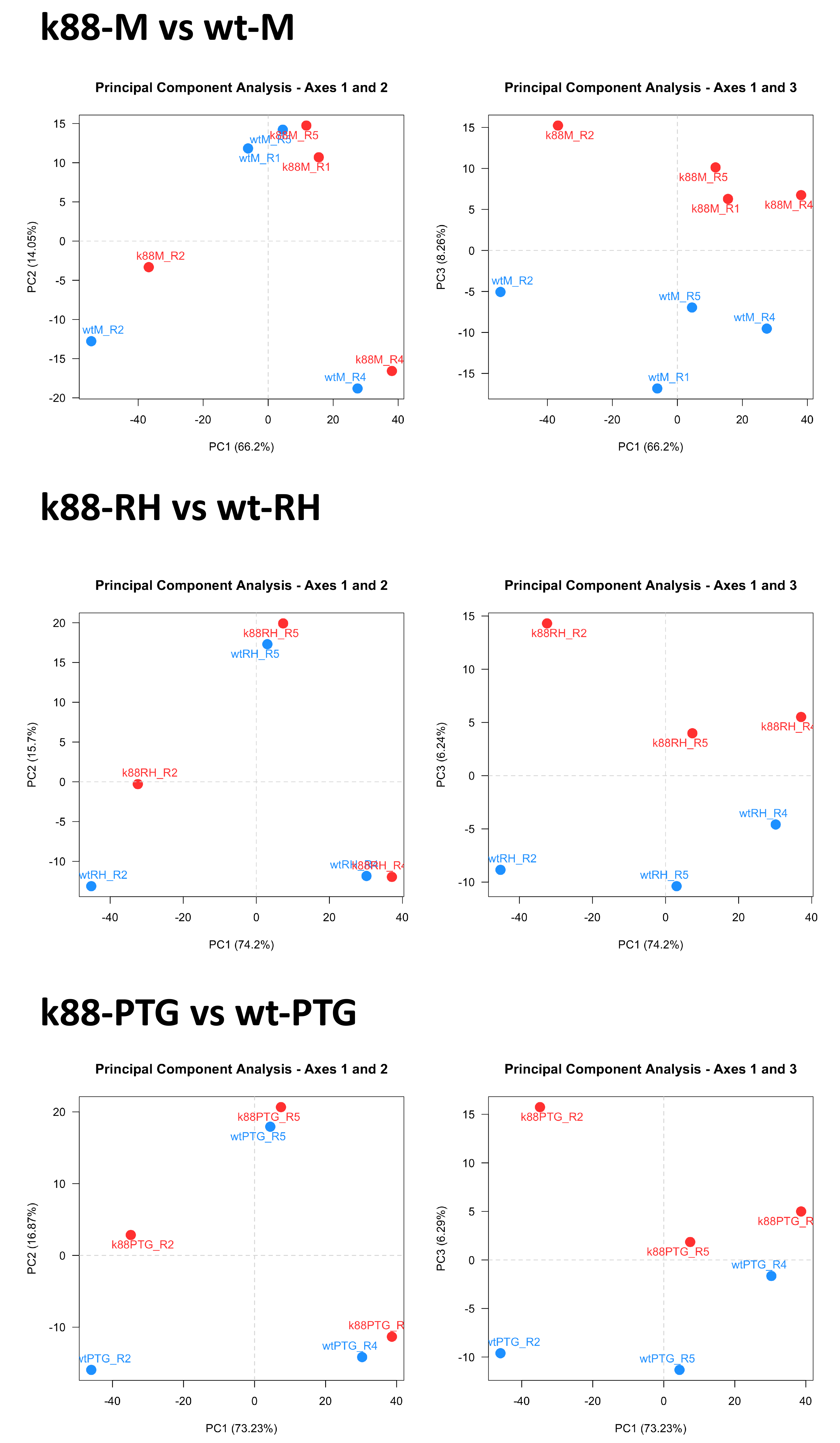

Supplement: Supplementary file 3 — Additional file 3. Principal component analysis (PCA) plots of mouse RNA-sequencing data reveal associations between samples: PCA plots of mouse transcripts for MyD88 KO BMDM versus wild type BMDM comparisons. [file 12864_2021_7437_MOESM3_ESM.tif]

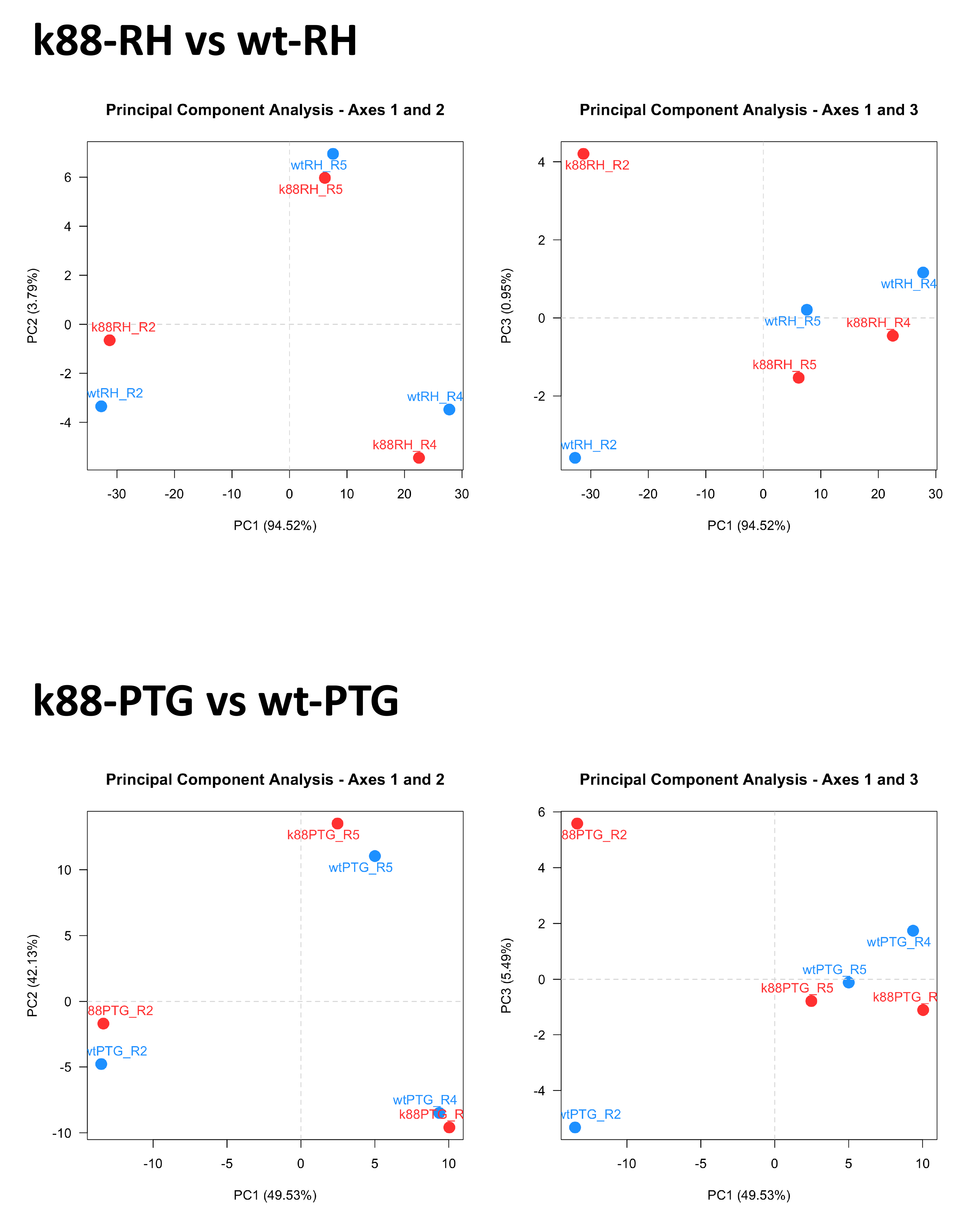

Supplement: Supplementary file 4 — Additional file 4 Principal component analysis (PCA) plots of T. gondii RNA-sequencing data reveal associations between samples: PCA plots of T. gondii transcripts for MyD88 KO (k88) BMDM versus wild type (wt) BMDM comparisons. [file 12864_2021_7437_MOESM4_ESM.tif]

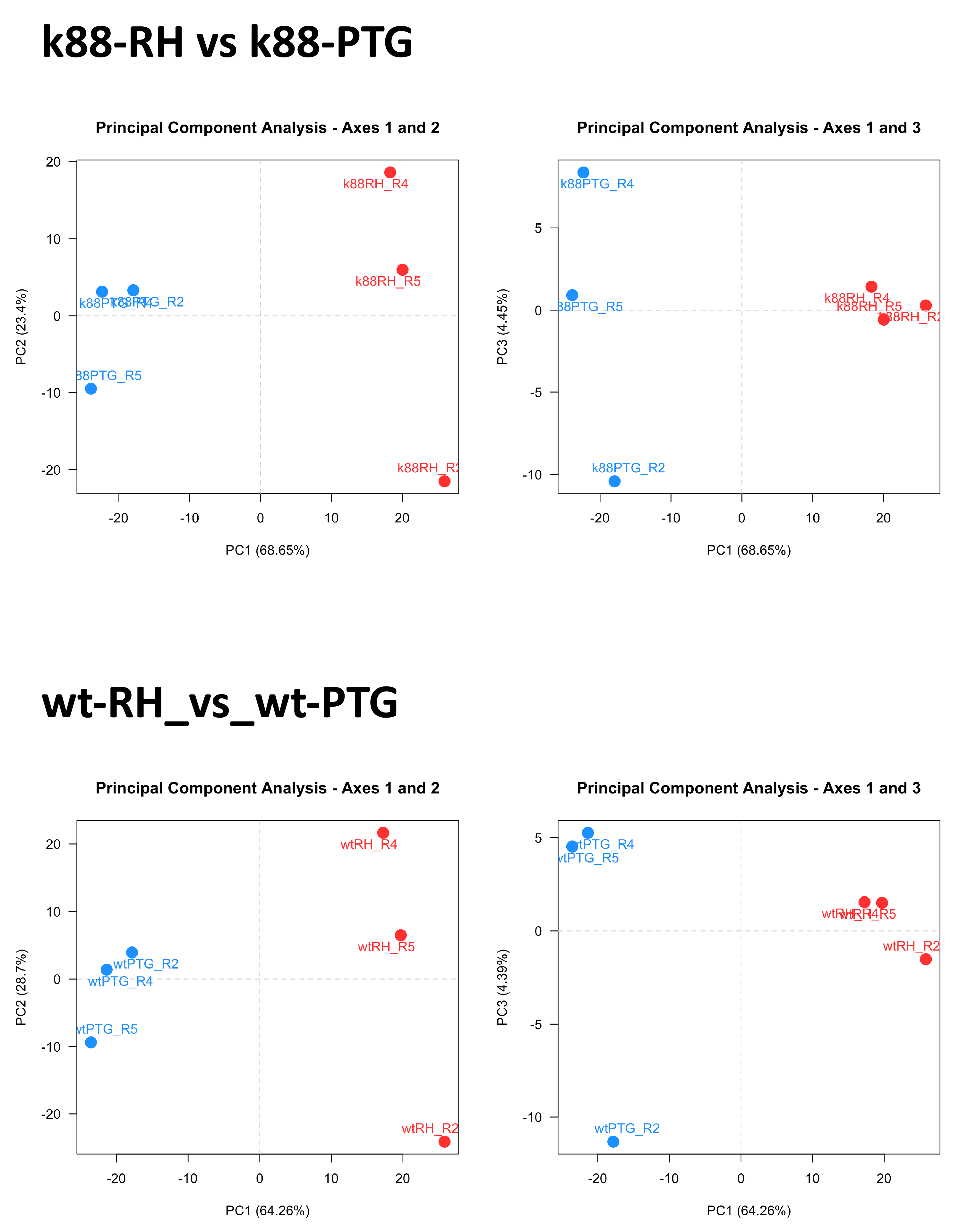

Supplement: Supplementary file 5 — Additional file 5 Principal component analysis (PCA) plots of T. gondii RNA-sequencing data reveal associations between samples: PCA plots of T. gondii transcripts for RH versus PTG comparisons. [file 12864_2021_7437_MOESM5_ESM.tif]

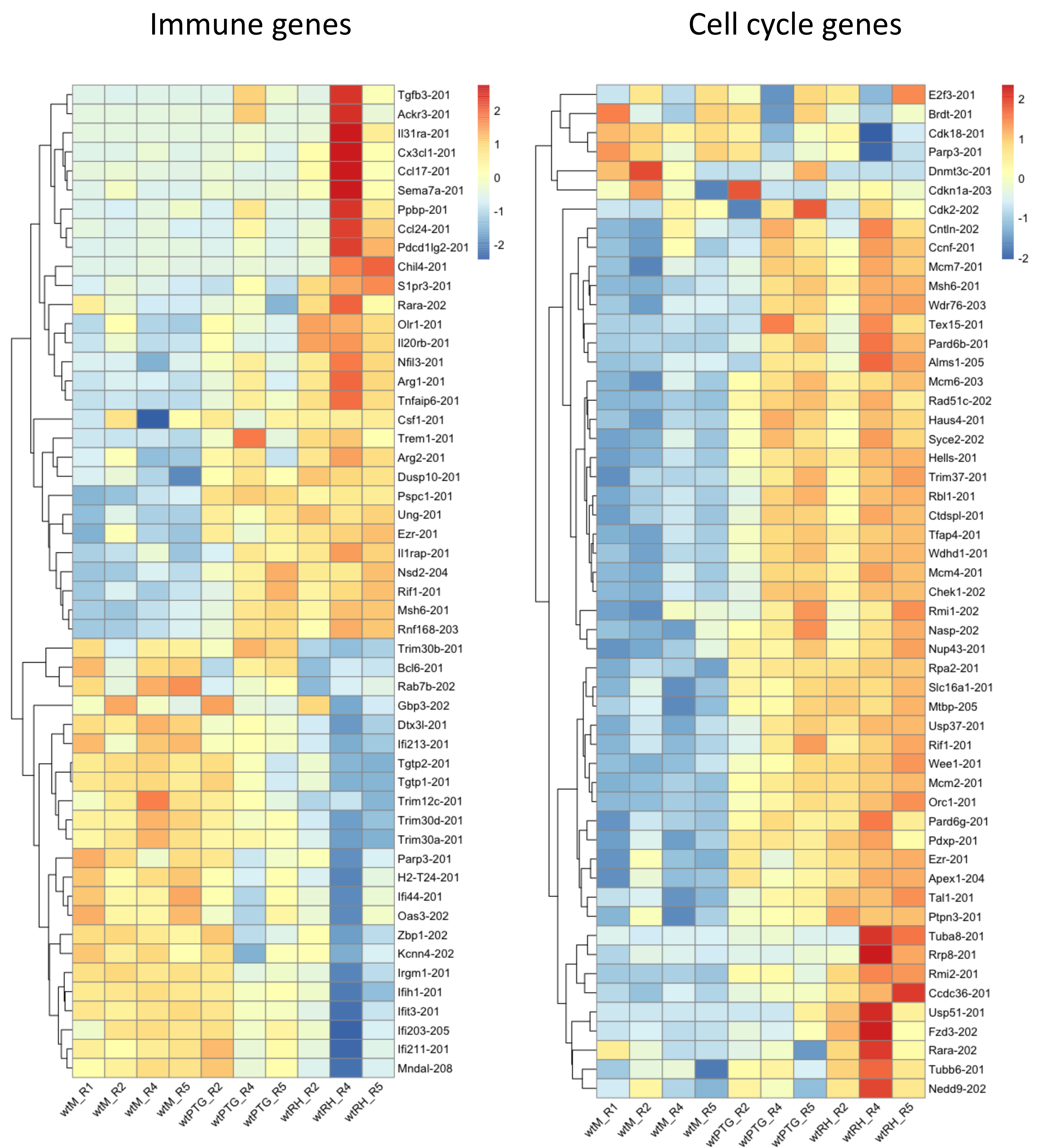

Supplement: Supplementary file 8 — Additional file 8. Heatmaps displaying individual replicates of differentially expressed immune and cell cycle genes in wildtype mice. Heatmaps displaying z-score transformed FPKM values of individual replicates among functionally related genes. [file 12864_2021_7437_MOESM8_ESM.tif]

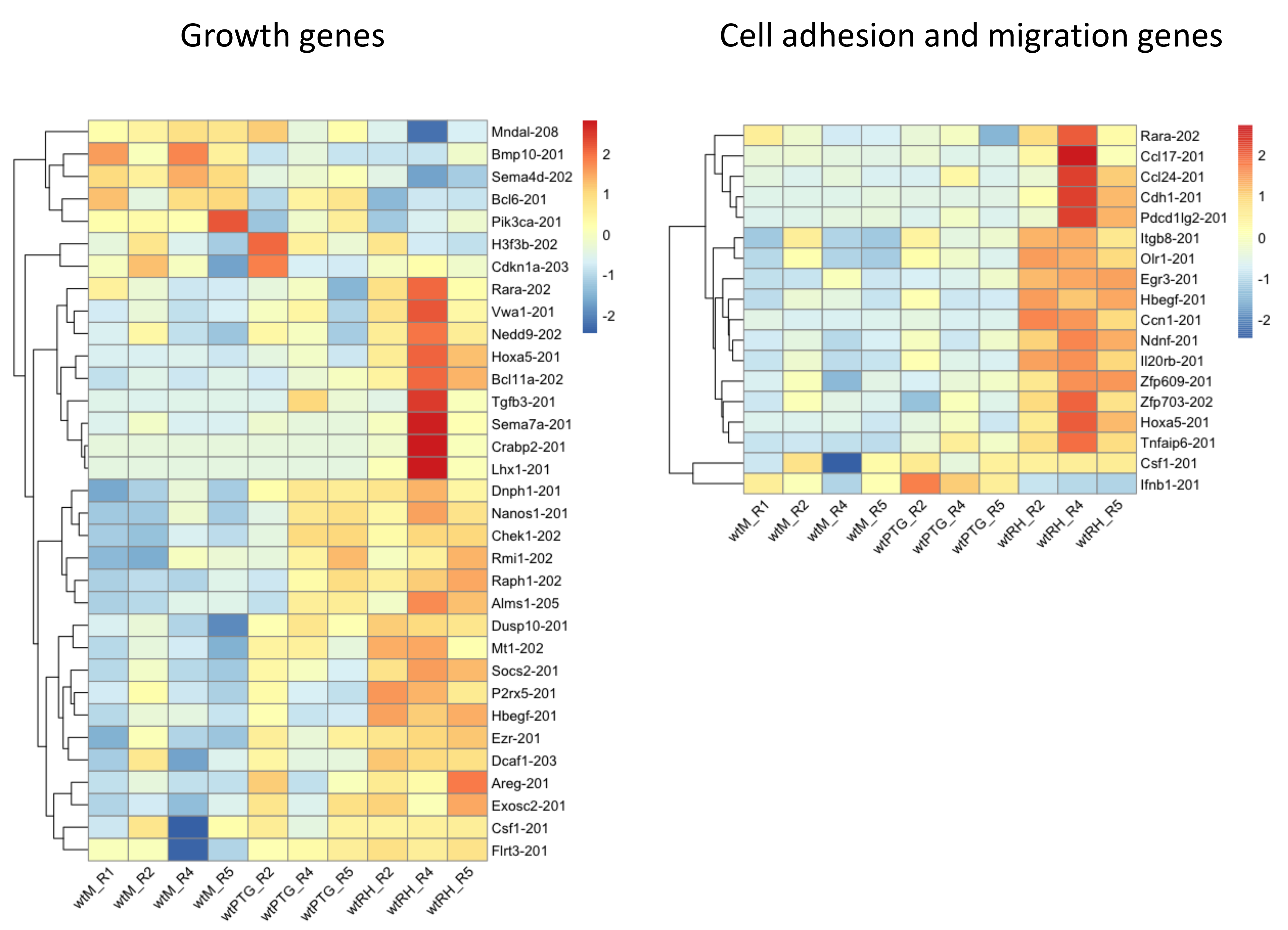

Supplement: Supplementary file 9 — Additional file 9. Heatmaps displaying individual replicates of differentially expressed growth, cell adhesion, and migration genes in wildtype mice. Heatmaps displaying z-score transformed FPKM values of individual replicates among functionally related genes. [file 12864_2021_7437_MOESM9_ESM.tif]

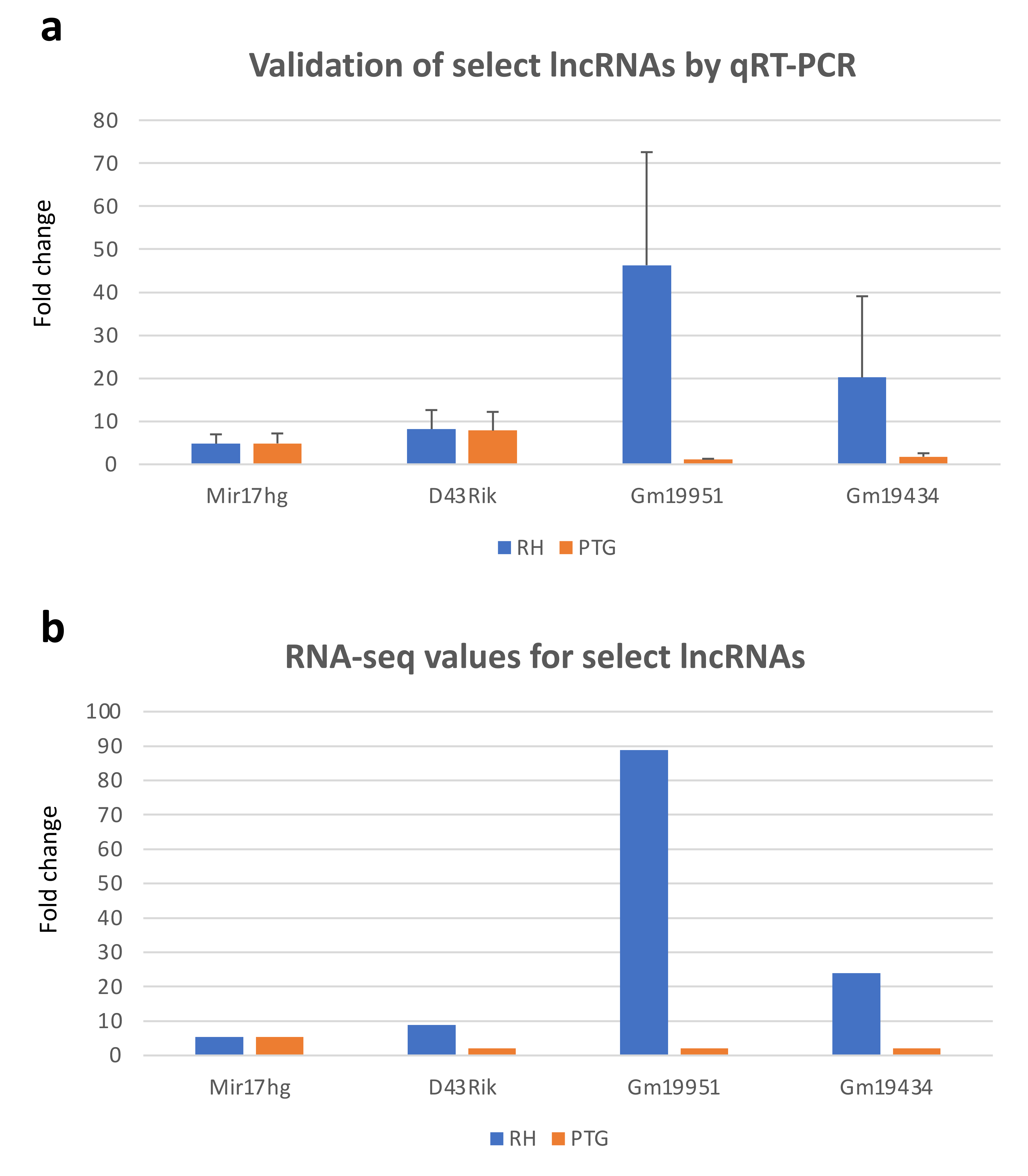

Supplement: Supplementary file 11 — Additional file 11 RNA-seq lncRNA data validation by qRT-PCR. RNA from mouse BMDM were collected 6 h after infection with either RH or PTG strains of T. gondii, and qRT-PCR was performed. Fold changes represent the comparison of infected samples to uninfected samples. (a) qRT-PCR fold change values for 4 lncRNAs. (b) RNA-seq fold change values for the same 4 lncRNAs for comparison purposes. Experiments were completed at least three times with BMDM from three separate mice. [file 12864_2021_7437_MOESM11_ESM.tif]

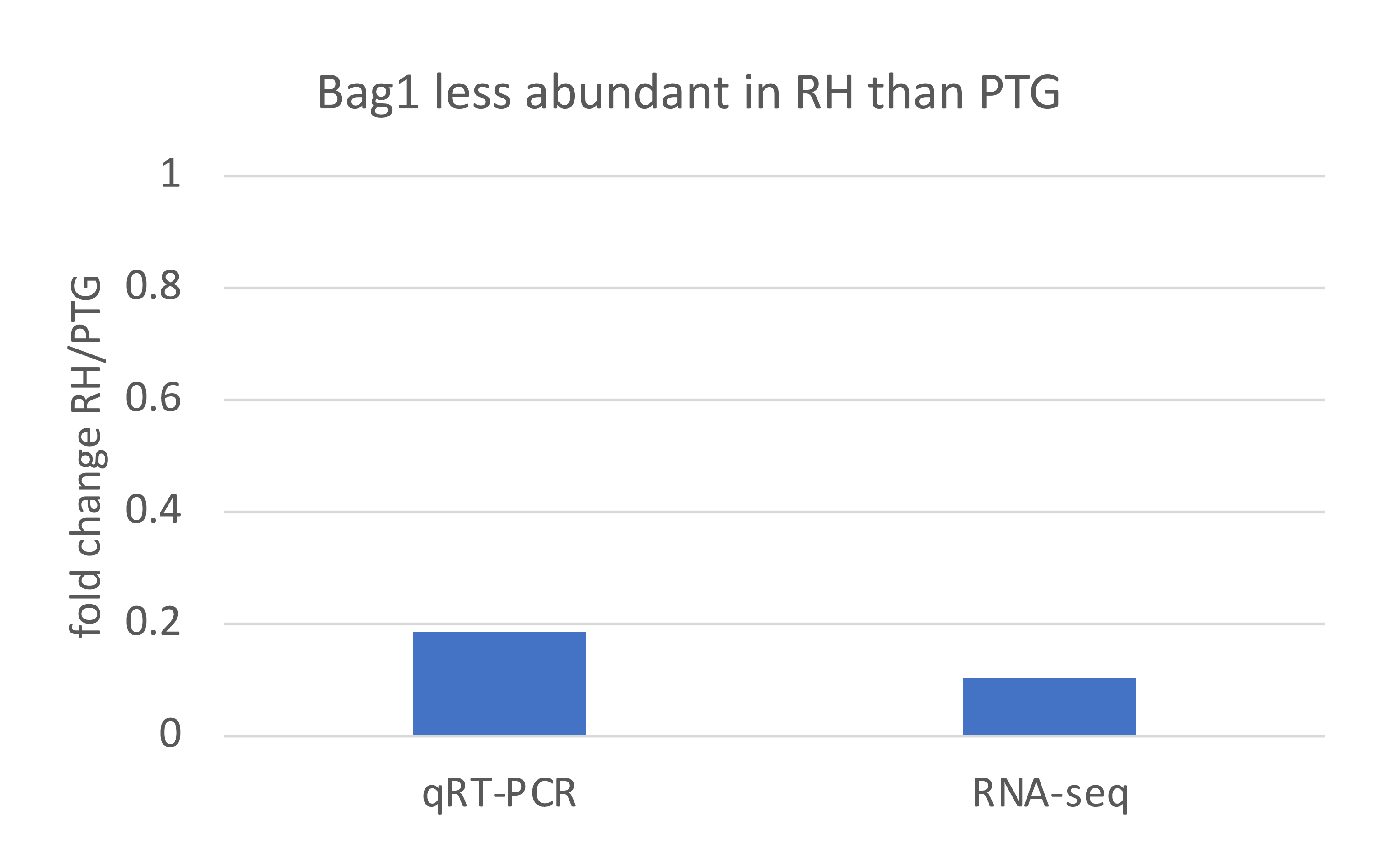

Supplement: Supplementary file 13 — Additional file 13 RNA-seq Toxoplasma gondii data validation by qRT-PCR. RNA from mouse BMDM were collected 6 h after infection with either RH or PTG strains of T. gondii, and qRT-PCR was performed. Fold changes represent the comparison of RH infected samples to PTG infected samples. RNA-sequencing and qRT-PCR data are displayed in one graph. Experiments were completed at least three times with BMDM from three separate mice. [file 12864_2021_7437_MOESM13_ESM.tif]
